# Heterogeneous drug tissue binding in brain regions of rats, Alzheimer’s patients and controls: impact on translational drug development

**DOI:** 10.1038/s41598-019-41828-4

**Published:** 2019-03-29

**Authors:** Sofia Gustafsson, Dag Sehlin, Erik Lampa, Margareta Hammarlund-Udenaes, Irena Loryan

**Affiliations:** 1 Translational PKPD Group, Department of Pharmaceutical Biosciences, Associate member of SciLifeLab, Uppsala University, Uppsala, Sweden; 2 Molecular Geriatrics, Department of Public Health and Caring Sciences, Uppsala University, Uppsala, Sweden; 3 Uppsala Clinical Research Center, Uppsala University, Uppsala, Sweden

## Abstract

For preclinical and clinical assessment of therapeutically relevant unbound, free, brain concentrations, the pharmacokinetic parameter fraction of unbound drug in brain (f_u,brain_) is commonly used to compensate total drug concentrations for nonspecific brain tissue binding (BTB). As, homogenous BTB is assumed between species and in health and disease, rat BTB is routinely used. The impact of Alzheimer’s disease (AD) on drug BTB in brain regions of interest (ROI), i.e., f_u,brain,ROI_, is yet unclear. This study for the first time provides insight into regional drug BTB and the validity of employing rat f_u,brain,ROI_ as a surrogate of human BTB, by investigating five marketed drugs in post-mortem tissue from AD patients (n = 6) and age-matched controls (n = 6). Heterogeneous drug BTB was observed in all within group comparisons independent of disease and species. The findings oppose the assumption of uniform BTB, highlighting the need of case-by-case evaluation of f_u,brain,ROI_ in translational CNS research.

## Introduction

The failure rate in CNS drug development is high and the struggle to find curative treatments for neurodegenerative disorders like Alzheimer’s disease (AD) is a costly mission for those companies undertaking it^1,2^. AD is the major cause of dementia worldwide, clinically manifested by progressive cognitive impairment, and with pathological hallmarks such as neuronal loss, brain amyloid beta (Aβ) deposits, and tau containing neurofibrillary tangles. Current AD management is only symptomatic and a global goal of finding an effective treatment or a way to prevent AD has been set to year 2025^3^. Early and precise diagnosis of AD, as well as appropriate clinical trial designs with sufficient justification for the choice of dose, are key aspects of successful drug development^4^. Proper dose selection in clinical trials is exceptionally challenging and it requires a knowledge-based confidence in processes governing pharmacological effect throughout CNS drug development. Accurate translation and evaluation of relationships between drug pharmacokinetic (PK) parameters and pharmacodynamic (PD) readouts from preclinic to clinic and from health to disease is therefore essential. The latter is valid not merely for CNS drug development but also for ample pharmacotherapy of multiple concomitant conditions in AD patients.

The clinical response of a drug, and hence treatment success, heavily relies on the achievement of adequate drug exposure at the target-site in the brain region of interest (ROI). Given the free drug hypothesis, it is only the unbound, free, drug that can interact with its target and elicit the pharmacological effect. Therefore, an assessment of unbound, rather than total brain concentrations is necessary for the accurate interpretation of drug exposure-effect or side effect relationships in the brain^5–9^. However, both the ability of a drug to reach sufficient brain exposure and the feasibility to measure relevant drug concentrations in brain, constitute major hurdles in CNS drug research, especially in humans^10,11^.

The achievement of steady state, unbound drug concentration in brain extracellular (interstitial) fluid is governed by various equilibration processes. Often the rate-limiting step of equilibration is dependent on the ability of unbound and unionized drug to cross the protective interface separating blood from brain, the blood-brain barrier (BBB). However, the disposition of drug within brain parenchyma, involving intracellular distribution, and specific and nonspecific brain tissue binding (BTB), could also contribute significantly to the attainment of steady state^10^. The drug’s propensity to bind nonspecifically, off target, to brain constituents, such as membrane lipids and proteins, is one of the quantitatively most significant processes that may locally influence the time to reach equilibrium, as well as half-life in brain^10,12–14^. The nonspecific binding is also the major contributor to the estimate of fraction of unbound drug in brain (f_u,brain_).

f_u,brain_ is a routinely studied neuropharmacokinetic (neuroPK) parameter used to compensate total drug concentrations in brain in preclinical PK studies to obtain unbound drug concentration estimates^10,15–17^. The equilibrium dialysis technique, utilizing brain homogenate, is widely used to determine f_u,brain_ and it allows for high-throughput screening of a wide range of compounds with differing physicochemical properties^16,18^. Positron emission tomography (PET) is a technique used for the study of drug PK and is highly translatable between species, with similar designs being applicable in both preclinical and clinical settings, however measuring only total concentrations. Recent studies in animals highlight the applicability of PET together with estimates of brain drug distribution, such as f_u,brain_, to generate unbound drug concentration estimates in brain^19,20^. PET is currently the only technique available for the use in humans to obtain neuroPK. Though, if PET is to be used in humans for the study of unbound drug concentrations and further BBB transport, it is essential that accurate compensation is made for drug intra-brain distribution in order to generate reliable estimates.

In drug development, f_u,brain_ is generally determined in one species, primarily rodents, with consequent use of the value in PK applications in both healthy volunteers and patients. The approach is based on findings from autonomus groups claiming species independence of the extent of drug BTB^21,22^. Most often, the whole brain (WB) tissue homogenate is used for f_u,brain_ determination. The methodology is grounded on an assumption of homogenous drug BTB throughout the brain. In a previous study in our group, non-homogenous BTB in brain regions of healthy rats was documented for antipsychotics, particularly, haloperidol, clozapine, risperidone and quetiapine^23^. Hence, in order to increase the confidence in validity of using extrapolation from preclinical species to humans, systematic investigations of drug BTB in humans and especially binding properties during disease conditions in discrete brain ROIs are needed.

While nonspecific drug-protein interactions occur, membrane partitioning, and hence drug-lipid interactions, has been suggested to dominate nonspecific drug binding in brain, resulting in a strong correlation between drug lipophilicity and BTB^24–26^. The numerical, morphological and functional diversity among cells of the CNS, as well as regional differences in brain lipid content in both rodent and human brain is a fact^27–32^. Given these region-specific characteristics, differences in drug regional BTB could oppose the uniform binding assumption. To our knowledge, there is so far only one study showing heterogeneous BTB in healthy rats^23^. It has also been suggested that regional differences in brain lipid composition are partly associated to selective neuronal vulnerability, where certain neuronal cells are more sensitive to age or stressors related to AD pathology^30^. In line with such hypothesis, studies have also shown altered lipid composition in specific regions of AD brain^31,33^. These alterations in lipid composition further imply that the extent of drug BTB and hence the f_u,brain_, could be altered in the brain of AD patients. This could in turn locally affect the fate of a drug within the brain and in particular the time it takes to reach steady state in brain. PK measures in healthy tissue might thus be misleading in the translation of data to clinical conditions.

The present study aims at establishing translational measures for improved predictions of therapeutic concentrations in AD patients, with the potential implication to aid dose selection of centrally acting drugs in early and late stage clinical trials, and in PET investigations. It is the first exploratory study aiming to provide enhanced understanding of drug BTB in various brain regions in health and disease from rodent to man, by elucidating the extent of BTB of five model drugs. The selection of the model drugs, donepezil, memantine, paliperidone, diazepam, and indomethacin was based on their differing physiochemical properties, attribution to different pharmacological classes and relevance for the management of AD or concomitant diseases.

## Results

### Pathological assessment of Aβ in human brain regions

**Figure 1:**
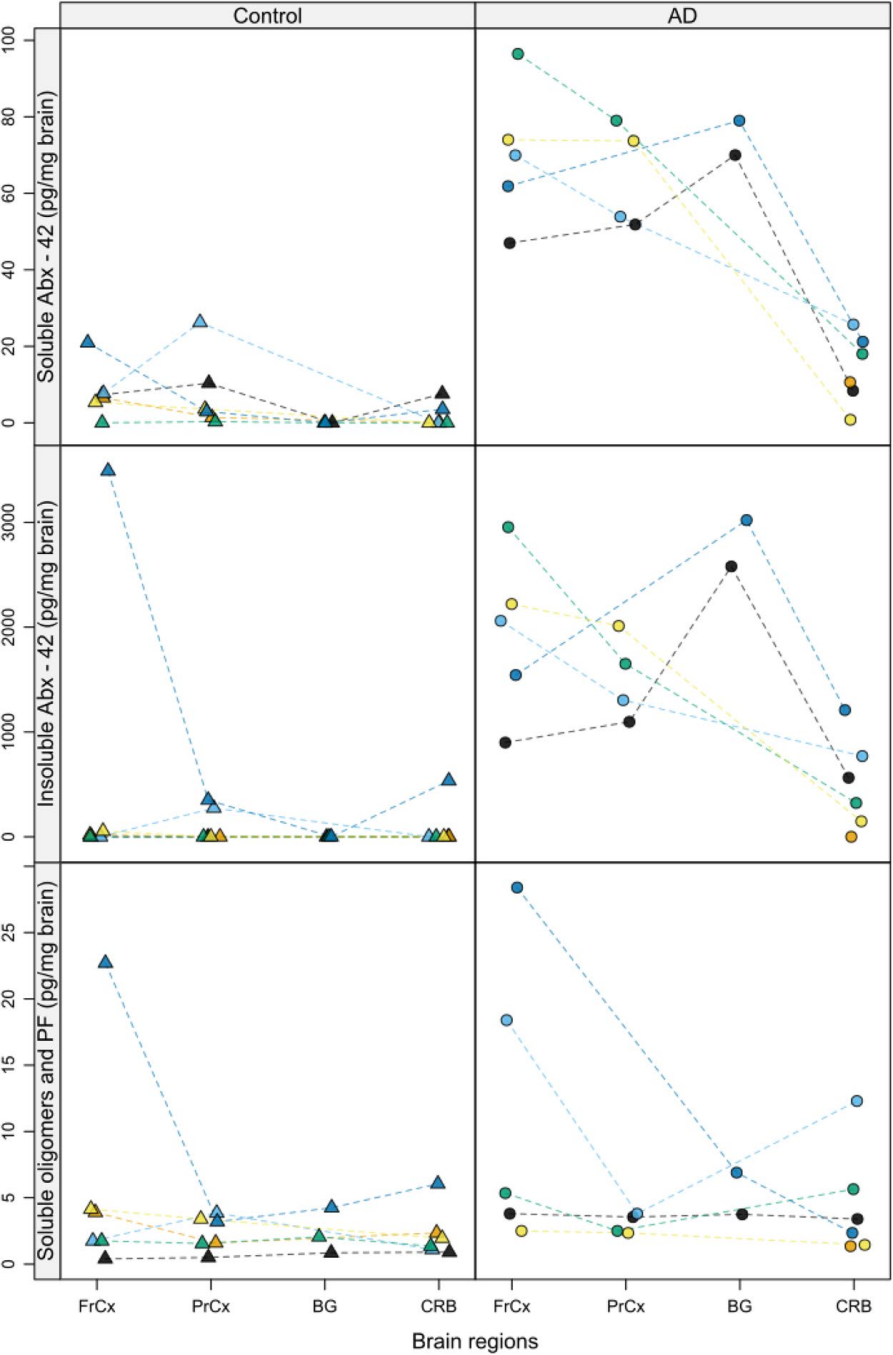
Regional concentrations of soluble and insoluble Aβx-42, and soluble Aβ oligomers and protofibrils in human post-mortem brain tissue. Triangles represent age-matched healthy donors (Group: Control) and circles represent AD donors (Group: AD). Each color is representative of one individual in the two groups respectively, with protein measures in available regions. Color coding of the control group, ID 1 (black), ID 2 (orange), ID 3 (light blue), ID 4 (green), ID 5 (yellow), ID 6 (blue). Color coding of the AD group, ID 7 (black), ID 8 (orange), ID 9 (light blue), ID 10 (green), ID 11 (yellow), ID 12 (blue). The interconnected lines are used for the ease of following regional measures in the same individual. FrCx, frontal cortex; PrCx, parietal cortex; BG, basal ganglia; CRB, cerebellum; PF, protofibrils.

Human post-mortem brain tissue from AD and control donors were included in the study based on clinical diagnosis and pathological findings, as documented by the Brain Bank at KI, where AD or dementia were confirmed or rejected in the respective groups (Table 1). Investigated brain regions included frontal cortex (FrCx), parietal cortex (PrCx), basal ganglia (BG), and cerebellum (CRB). In order to further verify the presence of Aβ, ELISA concentration measurements were performed showing noticeable differences in soluble and insoluble Aβx-42 between the AD and control groups, confirming pathology in the AD group (Fig. 1, Table S1). The levels of soluble and insoluble Aβx-42 were highest in FrCX and BG and lowest in CRB. Most of the AD donors showed values of soluble Aβ oligomers and protofibrils comparable to those observed in the control group, not exceeding 7 pg/mg of brain tissue. Only two AD donors, ID 9 and ID 12, showed elevated levels of soluble Aβ oligomers and protofibrils in FrCx, and one of these, ID 9, also displayed higher levels in CRB (Fig. 1, Table S1). ID 6 in the control group showed regional occurrence of AD related proteins with pronounced elevation of insoluble Aβx-42, and soluble Aβ oligomers and protofibrils, primarily in the FrCx. In the comparisons of BTB, ID 6 was kept in the control group based on catamnestic information confirming the lack of neurological symptoms. A sensitivity analysis showed that the inclusion or exclusion of ID 6 from the control group did not have any major impact on the conclusions of the statistical analysis performed on the regional BTB dataset (data not shown).

**Table 1:** Clinical and demographic data of control subjects without neurodegeneration and patients with Alzheimer’s disease. Autopsy material was acquired from the Brain Bank at Karolinska Institutet (BB@KI). AD, Alzheimer’s disease; CAA, Cerebral amyloid angiopathy; FrCx, frontal cortex; PrCx, parietal cortex; BG, basal ganglia; CRB, cerebellum; na, not available.

	ID no.	Gender	Age at death	Post-mortem interval (h)	Clinical diagnosis^a^	Pathological diagnosis^b^	CAA	Available brain regions			
								FrCx	PrCx	BG	CRB
**Control (n** = **6)**	ID 1	M	73	31	No impairment	No neurodegeneration	na	x	x	x	x
	ID 2	M	73	14	No impairment	No neurodegeneration	na	x	x	x	x
	ID 3	M	69	22	No impairment	No neurodegeneration	na	x	x	na	x
	ID 4	M	71	12	No impairment	No neurodegeneration	na	x	x	x	x
	ID 5	M	77	22	No impairment	No neurodegeneration	na	x	x	na	x
	ID 6	M	80	16	No impairment	No neurodegeneration	na	x	x	x	x
**Median**			**73**	**19**							
**AD (n** = **6)**	ID 7	F	78	12	AD	AD	Yes	x	x	x	x
	ID 8	F	87	49	Dementia	AD	na	x	x	x	x
	ID 9	F	89	34	Dementia	AD	Yes	x	x	na	x
	ID 10	F	83	32	Dementia	AD	Yes	x	x	x	x
	ID 11	F	81	26	Dementia	AD	na	x	x	x	x
	ID 12	F	83	8	No record	AD	Yes	x	x	x	x
**Median**			**83**	**29**							

^a^Based on the presence or absence of neurodegenerative disease.

^b^Pathological diagnosis according to the BB@KI.

### Quality assessment in the equilibrium dialysis experiments

To control for the quality of the equilibrium dialysis assay, the stability of tested drugs was determined as % remaining drug for each individual brain region (Eq. 3). In all experiments and for all studied drugs, the stability was 75–125% which is in the range of 70–130% (data not shown). The latter is a set range for the acceptable level of stability in equilibrium dialysis experiments^34^. Another crucial factor controlled for was the intra-day and inter-day variability. The variance was assessed by measuring f_u,brain_ of individual drugs, using identical whole rat brain homogenate every experimental day (three days for each drug). The experiments confirmed that the coefficient of variation in the inter-day assessment of f_u,brain_ was at an acceptable level below 20%, i.e., for diazepam (10.3%), donepezil (18.7%), indomethacin (5.76%), memantine (8.47%), and paliperidone (6.86%). Intra-day variability in all experiments was lower than 10%.

### Regional drug brain tissue binding in AD and healthy controls

**Figure 2:**
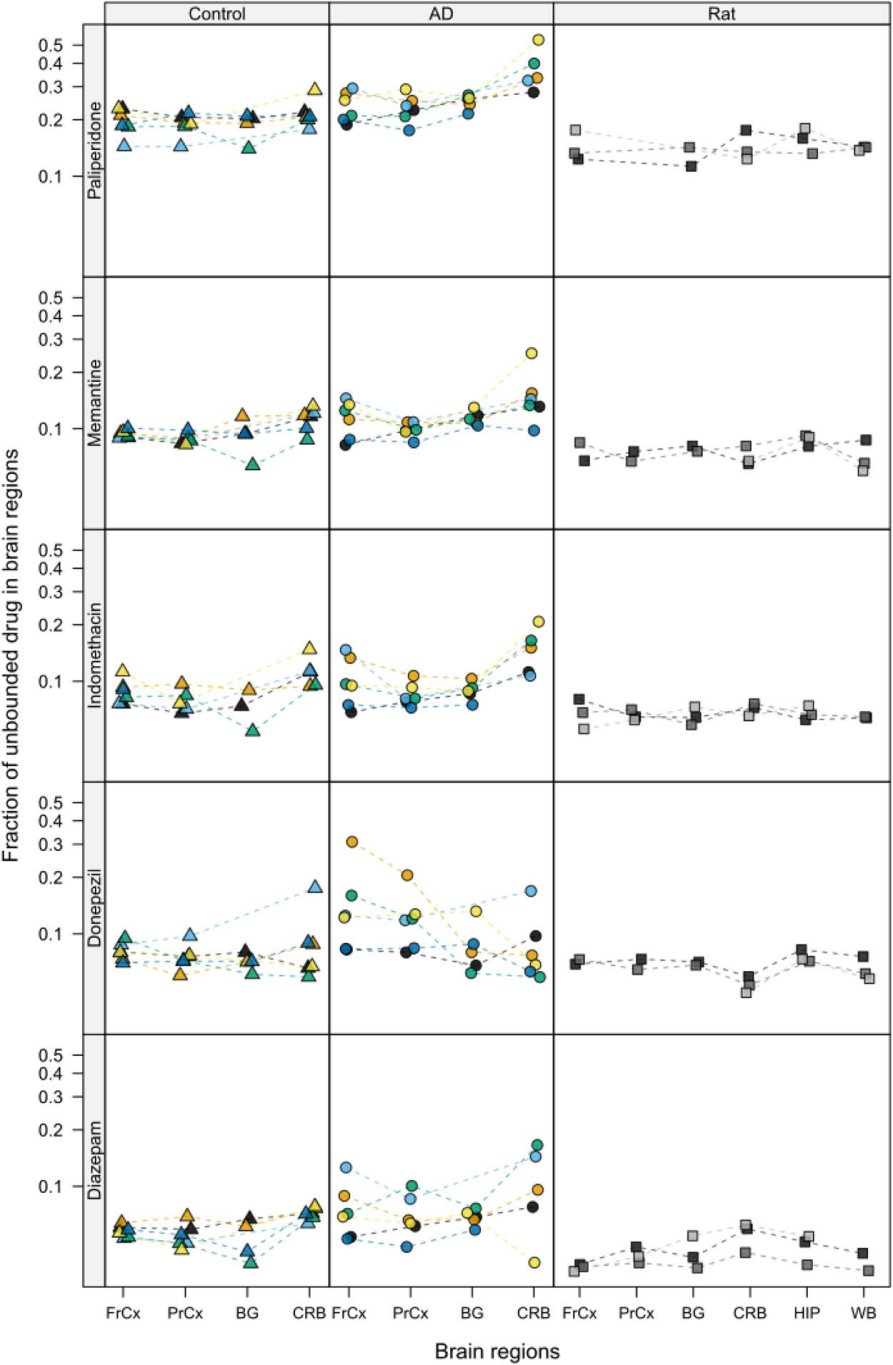
Regional drug brain tissue binding, presented as individual mean f_u,brain,ROI_ values (from technical replicates), in post-mortem brain regions of AD (Group: AD) and age-matched control donors (Group: Control), as well as healthy Sprague-Dawley rats (Group: Rat). Triangles represent controls, circles represent AD donors, and squares represents rats. For the human data, each color is representative of one individual in the groups, respectively, with f_u,brain,ROI_ measures in available regions. The gray scale is representative of pools consisting of rat brain tissue from 2–3 rats per pool. Color coding of the control group, ID 1 (black), ID 2 (orange), ID 3 (light blue), ID 4 (green), ID 5 (yellow), ID 6 (blue). Color coding of the AD group, ID 7 (black), ID 8 (orange), ID 9 (light blue), ID 10 (green), ID 11 (yellow), ID 12 (blue). The interconnected lines are used for the ease of following regional measures in the same individual. FrCx, frontal cortex; PrCx, parietal cortex; BG, basal ganglia; CRB, cerebellum; HIP, hippocampus; WB, whole brain; f_u,brainROI_, fraction of unbound drug in brain regions of interest.

All investigated drugs showed a high BTB, exceeding 80% in the control group (Fig. 2, Table 2). BTB was highest for diazepam and lowest for paliperidone. A between group comparison of the data, including the entire binding dataset of all five compounds, showed a difference in overall drug BTB between AD and controls (*p* = 0.009), however, not confirming the origin of the difference. Due to the innate demographic differences between the control and AD groups in age at death and post-mortem interval, their impact on regional drug BTB has been evaluated in the development of the linear mixed effects model used for between and within group comparisons (Table 1). The final model included brain region, pathology status (control or AD) and the interaction between them as fixed effects with further adjustment for post-mortem interval in hours.

**Table 2:** Descriptive statistics of fraction of unbound drug in brain ROIs (f_u,brain,ROI_) determined in post-mortem brain regions from control (Group: Control) and AD (Group: AD) donors, and rats (Group: Rat). Median values are based on the number of technical replicates (n), and mean values are based on the number of biological replicates (N). AD, Alzheimer’s disease; FrCx, frontal cortex; PrCx, parietal cortex; BG, basal ganglia; CRB, cerebellum; HIP, hippocampus; WB, whole brain; f_u,brain,ROI_, fraction of unbound drug in brain region of interest; SD, standard deviation from the mean.

Drug	Parameter	Control				AD				Rat					
		FrCx	PrCx	BG	CRB	FrCx	PrCx	BG	CRB	FrCx	PrCx	BG	CRB	HIP	WB
Paliperidone*	**Median**	0.198	0.183	0.192	0.207	0.216	0.224	0.249	0.350	0.138	na	0.128	0.143	0.150	0.151
	**1st quartile**	0.173	0.169	0.158	0.194	0.206	0.214	0.229	0.310	0.116	na	0.117	0.117	0.136	0.118
	**3rd quartile**	0.227	0.209	0.201	0.229	0.274	0.258	0.262	0.420	0.167	na	0.145	0.167	0.164	0.159
	**n**	18	18	11	18	17	16	12	15	7	na	5	9	4	7
	**Mean**	0.197	0.189	0.186	0.217	0.237	0.232	0.249	0.374	0.144	na	0.128	0.145	0.157	0.141
	**SD**	0.033	0.025	0.031	0.037	0.044	0.039	0.022	0.099	0.028	na	0.021	0.028	0.024	0.003
	**N**	6	6	4	6	6	6	5	5	3	na	2	3	3	3
Memantine	**Median**	0.092	0.086	0.093	0.116	0.117	0.103	0.111	0.142	0.073	0.068	0.077	0.078	0.090	0.071
	**1st quartile**	0.089	0.083	0.073	0.098	0.089	0.092	0.106	0.130	0.069	0.066	0.073	0.066	0.075	0.060
	**3rd quartile**	0.099	0.091	0.102	0.122	0.133	0.106	0.118	0.159	0.081	0.081	0.077	0.080	0.097	0.085
	**n**	18	18	11	18	17	17	13	17	4	5	5	9	9	7
	**Mean**	0.094	0.088	0.092	0.113	0.114	0.099	0.114	0.152	0.076	0.071	0.078	0.071	0.088	0.071
	**SD**	0.004	0.006	0.022	0.016	0.025	0.009	0.010	0.053	0.012	0.006	0.004	0.009	0.006	0.014
	**N**	6	6	4	6	6	6	5	6	2	2	2	3	3	3
Indomethacin	**Median**	0.088	0.083	0.071	0.109	0.095	0.080	0.089	0.116	0.065	0.064	0.066	0.076	0.068	0.065
	**1st quartile**	0.077	0.069	0.063	0.098	0.074	0.075	0.076	0.110	0.057	0.062	0.062	0.062	0.063	0.059
	**3rd quartile**	0.094	0.085	0.083	0.120	0.124	0.090	0.097	0.178	0.074	0.066	0.069	0.081	0.074	0.069
	**n**	18	15	7	15	18	17	13	15	7	10	10	9	10	6
	**Mean**	0.088	0.079	0.073	0.113	0.103	0.085	0.089	0.149	0.068	0.066	0.065	0.071	0.068	0.064
	**SD**	0.014	0.012	0.018	0.022	0.031	0.013	0.010	0.041	0.012	0.004	0.007	0.005	0.006	0.001
	**N**	6	5	3	5	6	6	5	5	3	3	3	3	3	2
Donepezil	**Median**	0.078	0.074	0.072	0.069	0.132	0.118	0.085	0.071	0.071	0.069	0.071	0.056	0.073	0.066
	**1st quartile**	0.072	0.066	0.066	0.065	0.084	0.088	0.068	0.065	0.069	0.067	0.070	0.045	0.072	0.058
	**3rd quartile**	0.090	0.082	0.077	0.074	0.152	0.132	0.090	0.086	0.074	0.076	0.072	0.061	0.080	0.075
	**n**	18	18	11	13	18	18	13	14	5	5	5	10	9	7
	**Mean**	0.081	0.076	0.071	0.069	0.147	0.122	0.086	0.071	0.071	0.069	0.069	0.054	0.076	0.065
	**SD**	0.009	0.012	0.008	0.065	0.085	0.045	0.028	0.065	0.003	0.006	0.002	0.005	0.006	0.009
	**N**	6	6	4	6	6	6	5	6	2	2	2	3	3	3
Diazepam	**Median**	0.057	0.052	0.046	0.072	0.068	0.064	0.069	0.101	0.036	0.042	0.043	0.059	0.049	0.040
	**1st quartile**	0.055	0.049	0.041	0.065	0.054	0.060	0.061	0.076	0.035	0.041	0.039	0.050	0.039	0.037
	**3rd quartile**	0.061	0.060	0.062	0.078	0.085	0.080	0.075	0.142	0.038	0.047	0.048	0.061	0.053	0.044
	**n**	18	18	11	18	17	17	13	15	8	10	9	10	10	6
	**Mean**	0.058	0.055	0.053	0.072	0.077	0.071	0.068	0.105	0.037	0.043	0.044	0.055	0.048	0.040
	**SD**	0.004	0.008	0.013	0.005	0.027	0.019	0.007	0.051	0.002	0.004	0.009	0.010	0.008	0.006
	**N**	6	6	4	6	6	6	5	5	3	3	3	3	3	2

^*^Rat f_u,brain,ROI_ values for paliperidone have been taken from a previous study by Loryan *et al*.^23^.

**Figure 3:**
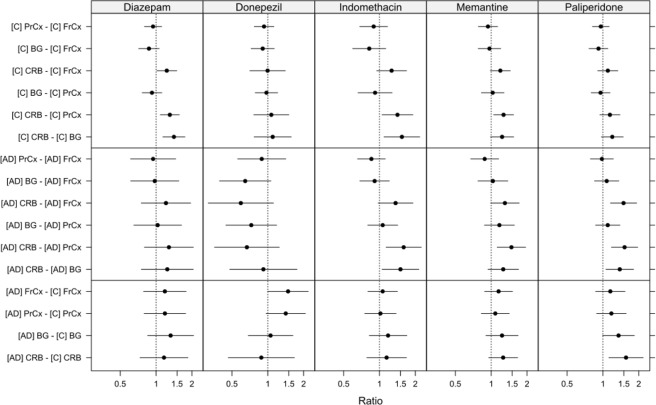
Caterpillar plot illustrating between region comparison of fraction of unbound drug in brain ROIs (f_u,brain,ROI_), within and between groups; controls [C], and Alzheimer’s disease patients [AD]. Data are presented as ratios of mean f_u,brain,ROI_ with 95% simultaneous confidence intervals. FrCx, frontal cortex; PrCx, parietal cortex; BG, basal ganglia; CRB, cerebellum. Value of 1.0, presented as a dotted line, indicates that mean values are the same.

Overall, the inter-individual and intra-individual variability in regional BTB was low in the control group (Fig. 2, Table 2). In a regional within-group comparison of the controls, donepezil and paliperidone displayed a uniform binding between the examined brain regions (Fig. 3, Table S2). However, the fraction of unbound diazepam, indomethacin and memantine was heterogeneous, with a minimum 1.3-fold higher f_u,brain,ROI_ value in CRB compared to PrCx, reflecting lower binding in the CRB. The f_u,brain,ROI_ of indomethacin and diazepam were similarly higher in CRB compared to BG (1.4-fold and 1.5-fold, respectively). Diazepam also displayed a higher f_u,brain,ROI_ in CRB compared to FrCx, indicating a pattern of lower binding of drug in CRB.

The variability in the AD group was noticeably higher than that in controls within each region, especially pronounced for donepezil and diazepam (Fig. 2). The regional within-group comparison of the AD donors, revealed that three out of the five studied drugs were characterized by a lower BTB in CRB compared to other brain regions, similar to the results in controls, while being more pronounced (Fig. 3, Table S2). Thus, memantine, indomethacin, and paliperidone showed higher f_u,brain,ROI_ in CRB compared to PrCx, all ratios being equal to or above 1.5. In addition, higher fractions of unbound indomethacin and paliperidone were documented in CRB compared to BG (1.5 and 1.4, respectively). In contrast, f_u,brain,ROI_ of donepezil in CRB was the lowest compared to other regions, reaching a median f_u,brain,ROI_ of 0.071 (Table 2), being significantly lower in CRB compared to FrCx (p = 0.025). Based on the regional within-group comparison, no statistically significant differences were observed in diazepam f_u,brain,ROI_, possibly due to the high inter-individual variability in discrete regions of the AD group (Fig. 3, Table S2). While the differences between regions were still within 2-fold, a pattern of lower binding in the CRB compared to other regions was observed for almost all compounds, except donepezil, in the regional comparisons in both the control and AD group.

Although the observed differences on a group level were modest or lacking, profound differences in drug f_u,brain,ROI_ between regions were apparent within the same individual, particularly in the AD group. For donepezil, four out of six AD donors showed significantly higher, up to 3.9-fold, f_u,brain,ROI_ values in FrCx and PrCx compared to CRB, while the other two donors displayed a very low variability, resembling binding profiles in the control group (Fig. 2).

Regional between group comparisons showed significant differences only for paliperidone and donepezil (Fig. 3, Table S2). Hence, a higher f_u,brain,ROI_ of paliperidone was observed in AD versus control CRB (ratio 1.6, 95% CI: 1.1–2.2, p = 0.002). Furthermore, higher f_u,brain,ROI_ in FrCx of AD patients compared to that in control subjects was observed for donepezil (ratio 1.5, 95% CI: 1.0–2.2, p = 0.033).

### Aβ and drug brain tissue binding relationship

**Figure 4:**
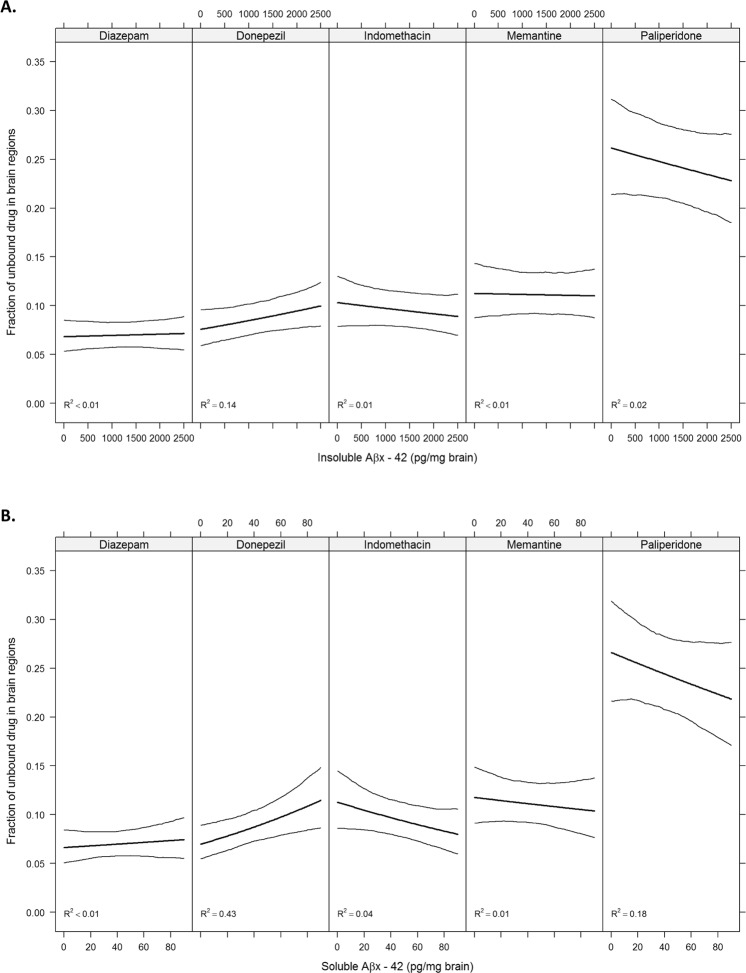
Fraction of unbound drug as a function of insoluble Aβx-42 (**A**) and soluble Aβx-42 (**B**). Bold lines denote mean fraction and thin lines denote 95% pointwise confidence intervals obtained through a parametric bootstrap procedure. R^2^ values denotes the fraction of variance explained by the proteins. The estimated association is a weighted average of the region-specific associations (See details in the Statistical Analysis section).

When the relationship between Aβ pathology and brain tissue binding of the five model compounds was investigated, no strong relationships were observed between binding and soluble or insoluble Aβx-42 (Fig. 4). The most prominent positive association was seen for donepezil f_u,brain,ROI_ and soluble Aβx-42 concentrations, with an R^2^ value of 0.43, indicating that 43% of the variability in regional BTB of donepezil could be explained by soluble Aβx-42 (Fig. 4B). No relationship was observed between soluble Aβ oligomers and protofibrils and f_u,brain,ROI_ for any of the compounds (data not shown). However, the concentration range of oligomers and protofibrils was very narrow and the highest concentrations were only present in 3 out of 12 individuals, possibly hampering the analysis.

### Regional drug brain tissue binding in rats

**Figure 5:**
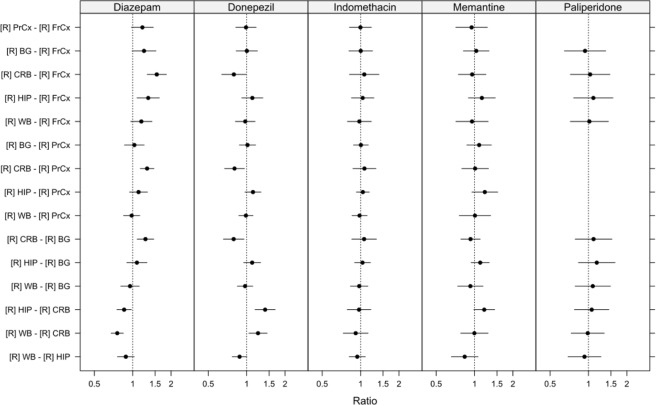
Caterpillar plot illustrating between region comparison of fraction of unbound drug in brain ROIs (f_u,brain,ROI_) in healthy Sprague-Dawley rats. Data are presented as ratios of mean f_u,brain,ROI_ with 95% simultaneous confidence intervals. FrCx, frontal cortex; PrCx, parietal cortex; BG, basal ganglia; CRB, cerebellum; HIP, hippocampus; WB, whole brain. Value of 1.0, presented as a dotted line, indicates that mean values are the same.

When investigating drug tissue binding in brain regions of healthy Sprague-Dawley rats, the f_u,brain,ROI_ values were similar to humans, with the lowest fractions for diazepam and the highest for paliperidone (Table 2, Fig. 2). In the between region comparison using a linear mixed effects model, it was shown that diazepam had a higher f_u,brain,ROI_, and hence lower binding, in CRB compared to all other regions as well as whole brain (WB) (Fig. 5, Table S3). Interestingly, the opposite was observed for donepezil, which displayed lower f_u,brain,ROI_ values, thus higher binding, in CRB compared to all other regions and WB measurements. The binding of diazepam and donepezil did not differ between other regions and no differences in binding were observed between regions for indomethacin, memantine and paliperidone (Fig. 5, Table S3).

### Relationship between rat and human drug brain tissue binding

The relationship between f_u,brain,ROI_ in healthy Sprague-Dawley rats and f_u,brain,ROI_ in control subjects or AD patients were investigated (Fig. S1). Overall, the rat data showed higher binding than control subjects, while being within 2-fold of the line of identity, for all studied drugs and investigated brain regions. However, the comparison of regional BTB in rats and AD patients revealed drug specific and region specific discrepancies. For instance, donepezil f_u,brain,ROI_ in rat FrCx overestimated the binding in FrCx of AD patients. Remarkably, for paliperidone, memantine, indomethacin and diazepam, the f_u,brain,ROI_ in CRB of AD patients was on average twice as high as compared to that in rat CRB.

## Discussion

Homogenous tissue binding of compounds within the brain and between species, as well as between health and disease is often assumed. However, the current study demonstrates that regional differences in drug BTB do exist within and between species and AD pathology. BTB differences were primarily observed between different brain regions in rats and in humans, independent of AD pathology. However, discrete differences were also apparent in the same regions of controls and AD patients. In addition, BTB in rats was generally shown to overestimate the binding of drugs in human brain regions. This was especially pronounced for CRB estimates in rat compared to AD patients, where the fraction unbound in rat was half that in AD patients for four out of the five drugs investigated. Hence, the current investigation of translational aspect of BTB between rodents and humans, and particularly AD patients, brings important insight into the understanding of CNS drug disposition in humans.

Considering the high attrition rates in CNS drug development, effort must be put into developing and optimizing translational tools for better predictions of unbound brain concentrations in humans and patients. In this regard, PET is a unique technique allowing *in vivo* biodistribution studies of brain total drug concentrations in preclinical species as well as in humans. Recent studies in pigs and rats have applied nonspecific binding estimates and brain drug distribution measures from WB to determine unbound drug concentrations from total concentrations acquired through brain PET imaging^19,20^. However, the present study refines the need of using region-specific data for improvement of target-site unbound concentration estimates.

The majority of brain PET imaging studies use the reference tissue model (RTM) or some variant of it, e.g. the simplified reference tissue model (SRTM), for quantification of data, where frequently *a priori* CRB is used as the reference region^35^. One of the main assumptions in RTM is that the reference and the target tissues have the same nonspecific BTB. Using mathematical simulations, Salinas *et al*. showed introduction of a significant bias in brain PET data when that assumption is violated^36^. The current findings of a repetitive pattern of generally lower BTB in human CRB compared to other regions, for four out of five compounds in AD patients and controls, violates the assumption of a uniform BTB. In addition, extensive intra-individual variability in regional BTB was observed in the AD group, which was highly pronounced for donepezil with up to 3.9-fold differences in BTB between regions of the same patient. Thus, the findings highlight the need of case-by-case evaluation of regional BTB, especially in disease conditions prior to applying RTM in CNS drug research.

In addition to challenges in CNS drug development, pharmacotherapy of various comorbidities in patients with AD is currently poorly investigated. Among them is pharmacological management of psychosis in AD patients, in whom exaggerated side effect profiles are reported^37,38^. There are few attempts to select optimal doses for personalized treatment of specific target populations via in-depth evaluation of the relationship between the target occupancy and clinical efficacy of antipsychotics^39,40^. Remarkably, the significant contribution of PK changes, including transport across the BBB, has been proposed. In this regard, the finding of an average 1.5-fold lower BTB of paliperidone in CRB of AD patients compared to frontal and parietal cortices may spread light on the region-specific changes occurring in the AD patient population. The latter may not be a sole attribute of paliperidone but also other antipsychotics, which will require further evaluation. This is mainly supported by our previous study in rats, confirming the existence of regional heterogeneity in both BBB transport and BTB of several antipsychotics^23^.

The documented dissimilarities in regional BTB found in the present study provide essential contributions to the understanding of CNS disposition of small drugs in different populations and especially AD patients with known changes in brain parenchymal neuroanatomy. Yet, the underlying mechanisms of the observed regional BTB heterogeneity of the investigated drugs requires further investigation. Numerous scientific reports address the intricate mechanisms of drug tissue binding, among them the role of melanin^41^, actin and myosin^42^, tubulin^43^ and primarily phospholipids^44,45^. Drug binding to brain tissue is mainly dependent on interactions with membrane lipids, which is shown to exceed protein binding^24,25,46^. Drug physicochemical properties and lipid composition determine drug interaction to or distribution within the lipid membranes^12,47^. While lipidomic data seem to vary due to technical diversity and tissue preservation, much of the present literature, on both rodents and humans, indicate a non-uniform lipidome throughout the brain, which could affect the binding pattern of drugs in specific regions^27,28,31,33^. Cholesterol makes up a large bulk of the brain lipidome while glycerophospholipids comprise the largest group of phospholipids^31,33^. Glycerophospholipids, together with sphingolipids, are important players in drug binding^24,25,44,45^. In a study by Chan and coworkers, differences in lipid composition were observed between brain regions of AD and control donors^33^. However, in their study predominant differences in AD compared to controls primarily involved less abundant lipids with potential roles in synaptic function and neurotransmission rather than lipids with higher abundance in cellular membranes and white matter tracts^33^. On the contrary, the more abundant lipids will most likely be of higher quantitative importance for drug binding in different regions. Sphingomyelin, being the largest class of sphingolipids, was slightly increased in the prefrontal cortex of AD patients, while unchanged in other investigated regions^33^. Lipids like phosphatidylcholine and phosphatidylethanolamine are dominating the glycerophospholipids, and were found to be unchanged between AD and controls^33^. These findings are in support of the present results, where moderate differences were observed between AD and controls. Other studies of brain lipid alterations in AD have reported both decreased and unchanged levels of glycerophospholipids, as well as increased or decreased levels of sphingolipids^31^. However, the choice of brain regions and analytical methods differ between these studies, which could render inconclusive data.

The inter-individual variability within regions was more pronounced in the AD group compared to controls in the present study, and especially compared to rat data. Giving the resemblance of the extent of BTB data in the control group, this variability is thought to be related to pathological diversity in the AD group. Importantly, our current results show that binding estimates in rats will most likely overpredict the binding of drugs in humans, and mask the variability in binding observed within the same region in the AD group. In a study of lipids in rat brain, Chavko *et al*. found phospholipid concentrations in the order of 43 mg/g wet brain, which was higher than that previously reported for humans, in the range of 17–23 mg/g wet brain^28^. This is in support of the current results, where rat brain binding is higher for all drugs compared to humans, indicative of a higher binding to lipids. However, a concern of human versus rat studies is the post-mortem interval, which might affect lipid concentrations and composition, and which is usually much longer in humans compared to preclinical studies. In addition, studies on BTB assessment performed using animal models mimicking amyloid beta and alpha-synuclein pathology did not observe significant differences compared to wild type mice, indicating potential neuroPK differences in relation to patients^48,49^. In particular, Gustafsson *et al*.^48^ studying BTB of diazepam and paliperidone in rostral and caudal brain regions revealed no BTB differences in amyloid beta pathology that are not recapitulated in AD patients (Figs 2 and 3).

Brain pathology was verified in the AD donors, with regional manifestations of soluble and insoluble Aβ42, with the highest concentrations in the FrCx and BG, and with the lowest accumulation in the CRB, which is in line with previous documented disease progression^50,51^. When put in relation to drug binding, we did not observe any strong relationship between Aβ42 and fraction unbound. Interestingly, both soluble and insoluble Aβ42 were able to separate AD from controls, while the levels of soluble Aβ oligomers and protofibrils only peaked in the FrCx of two individuals in the AD group and the rest of the AD patients showed comparable values to controls. An emerging theory in Aβ pathology stress that it is the oligomers and protofibrils that are the most toxic species of Aβ compared to insoluble Aβ, primarily representing Aβ deposited in plaques, or the monomeric species of Aβ, included in the soluble portion of Aβ42 in the present study. As such, oligomers and protofibrils could possibly exert the highest impact on membrane integrity and hence lipid composition and drug binding^52,53^. For the interest of further studies, it would therefore be essential to include individuals with more profound levels of this toxic Aβ species.

The human tissue samples used in previous studies evaluating species dependency of binding, were not well defined or reside from one region or one individual only^21,22,49^. The current study employed aged human brain from controls without neurodegenerative disease as well as AD patients, with further comparison of data to brain tissue from young rats commonly used in preclinical DMPK analysis. This study also involved up to four different regions from the same individuals, which allows studying intra-individual variability between regions. By including brain tissue samples from several individuals, we were also able to describe the inter-individual variability that was shown to be especially pronounced in the AD group. Despite the limitations of the clinical dataset and tissue availability, this study is central as it represents a first attempt to characterize regional BTB in humans. However, a more optimal age and gender distribution within the AD and control group would have been more favorable, as these factors may influence regional lipid composition and binding.

Based on the obtained results several recommendations could be provided for both preclinical and clinical neuroscientists. The present findings support the use of rat WB homogenate for the assessment of regional BTB of small molecular drugs with further extrapolation to humans without neurological impairment, bearing in mind the general overestimation in f_u,brain,ROI_. The latter has been also observed by Di *et al*. when comparing BTB between healthy Sprague-Dawley rat whole brain and healthy male occipital cortex^21^. However, it is not recommended to use rat WB f_u,brain_ as a surrogate of regional BTB in AD patients to compensate total drug concentrations for estimation of unbound target-site concentration, since the potential bias will most likely be larger than the two-fold acceptance in industry. These findings argue strongly for the consideration of region-specific adjustment of BTB in AD patients in PK/PD modeling and PET brain imaging.

In summary, the current study confirms heterogeneous, regional brain tissue binding in both healthy rats and humans. AD patients overall have lower BTB compared to age-matched controls, which was even more pronounced compared to healthy rats. These findings will force revisiting of applications where homogenous BTB is assumed, including but not limited to brain PET imaging and PK/PD modeling. The present conclusions may also foster CNS drug development and improve dose adjustment for AD patients by providing knowledge-based evidence of variable regional BTB of small molecular drugs.

## Materials and Methods

Data and associated protocols will be available to readers without undue qualifications in material transfer agreements. Bioanalysis of samples using LC-MS/MS method was performed in accordance with the guidelines from FDA^54^.

### Study design

The pre-specified hypothesis of the study was that drug distribution governed by nonspecific brain tissue binding in various brain regions differs in AD patients in relation to region-specific and disease-specific changes. The overall objective of this pioneering exploratory study was therefore to investigate regional brain tissue binding of five model drugs: donepezil, memantine, paliperidone, diazepam, and indomethacin (Table S5) in post-mortem tissue from AD patients and age-matched controls, while also verifying pathological hallmarks of AD in the studied brain regions from the same individuals. In addition, to test the validity of the use of WB tissue binding data obtained from rats as a surrogate of human BTB, comparison of generated human data with rat BTB was performed.

Prior to starting the experiments several preventive measures were taken to allow the most accurate assessment of BTB in human brain regional material. In particular, the commonly used method of equilibrium dialysis was optimized for the selected compounds to use the minimally required volume of brain homogenate, which was 100 µL. A robotic automated liquid handling system was used for sample preparation to assure the most accurate and unbiased sample preparation. The intra- and inter-day reproducibility of the results, obtained using technical replicates from the same biological matrix of rat brain tissue, showed a coefficient of variation below 10%. Based on the evidence supporting low inter-individual variability obtained in the studies using healthy rats (own observations), as well as preexisting knowledge on lack of species differences in brain tissue binding^34^, the obtained mean values with the standard deviation from the mean have been used to calculate the sample size in each group. Minimally required per-group sample size for a two-tailed t-test study was 6, given the probability level (α = 0.05), the anticipated effect size (Cohen’s d = 1.5), and the desired statistical power level (0.8)^55^. Similar inter-individual variability was assumed in both the AD and control group, because of the lack of any publicly available information on BTB in AD patients. During the course of the study, it became abundantly clear that the BTB in AD patients has much higher inter-individual variability than the control group and rats. Hence, this may indicate that the pre-selected sample size was underestimated. In this regard, the power of the study could be lower than claimed implying that only large effects can be detected, and negative results cannot be reliably interpreted.

The decision to not measure total serum albumin concentration in the brain tissue material has been made based on the reported investigation by Longhi *et al*. that have found neglected effect of serum albumin on BTB^56^. It is important to highlight that according to prior information, among the studied drugs only indomethacin has a plasma protein binding exceeding brain tissue binding on average by 5-fold. The latter means that in theory, the differences in the volume of residual blood in human brain tissue may impact assessment of BTB of indomethacin, i.e. the bigger is residual blood volume the lower is f_u,ROI_.

### Chemicals

Paliperidone and 9-hydroxyrisperidone-D4 (paliperidone-D4), donepezil hydrochloride, and memantine hydrochloride were purchased from Sigma-Aldrich (Stockholm, Sweden), and indomethacin was purchased from Fluka (Stockholm, Sweden). Diazepam was obtained from Apotek Produktion & Laboratorier AB (Stockholm, Sweden). Donepezil-D5 hydrochloride and memantine-D6 hydrochloride were purchased from Bio-Techne (Abingdon, United Kingdom), diazepam-D5 from Cerilliant (Round Rock, TX, USA) and indomethacin-D7 from Clearsynth (Budapest, Hungary). Acetonitrile and formic acid were obtained from Merck (Darmstadt, Germany). All other chemicals and reagents used in experiments were of analytical grade. The water used was deionized in-house and purified with a Milli-Q Academic system (Millipore, Bedford, MA, USA).

### Human brain autopsy material

Frozen tissue material from patients with confirmed AD (n = 6) and age-matched controls with no confirmed neurodegenerative condition (n = 6) were obtained from the Brain Bank at Karolinska Institutet (BB@KI), Sweden (ethical approval 2011/962–31/1). Informed consent from tissue donors was obtained by BB@KI. The samples were stored at −80 °C. All handling and experimentation using human post-mortem samples were approved by the regional Ethical Review Board in Uppsala (ethical approval 2014/268). Demographic data, clinical and pathological status, and brain region availability are presented in Table 1.

### Experimental animal brain material

Brain tissue was harvested from 25 male Sprague-Dawley rats, 280–320 g (Taconic, Lille Skensved, Denmark), for inclusion in the BTB measurements. The animals were group housed at 20–22 °C under a 12 h light/dark cycle, with *ad libitum* access to food and water. Under deep anesthesia using isoflurane (Baxter Medical AB, Kista, Sweden), the rats were decapitated and brains were collected and immediately placed on ice. The brains were dissected in a sagittal plane by the longitudinal fissure. ROIs such as FrCx, PrCx, CRB, BG, and hippocampus (HIP) were sampled according to Glowinski and Iversen^57^. The tissue was weighed and samples were stored at −20 °C. All animal procedures were performed in accordance with the guidelines of the Swedish National Board for Laboratory Animals and were approved by the local Animal Ethics Committee in Uppsala, Sweden (ethical approval C189/14).

### Aβ quantification

Brain concentrations of soluble and insoluble Aβ, as well as soluble Aβ oligomers and protofibrils, were determined in human brain regions as described previously, with some modifications^58^. In order to obtain a preparation of soluble protein, 10–15 mg of each tissue sample, as given by Table S4, were diluted at a 1:10 (w:v) ratio in Tris buffered saline, pH 7.6 (TBS, 20 mM Tris, 137 mM NaCl) with complete protease inhibitors (Roche, Basel, Switzerland). The tissues were homogenized in Precellys CK14 0.5 mL tubes containing 1.4 mm ceramic beads, using a Precellys Evolution homogenizer (Bertin Technologies, Montigny-le-Bretonneux, France). The samples were centrifuged at 16 000 *g* for 1 h at 4 °C, and the supernatant was collected and stored at −20 °C until analyzed. To acquire a preparation of insoluble proteins, as found in amyloid plaques, the TBS pellets were re-homogenized in 70% formic acid at a 1:10 w:v ratio, followed by centrifugation as above. The supernatant was stored at −20 °C until use. For ELISA measurements of soluble and insoluble Aβx-42, 96-well plates were coated with 1 µg/mL of polyclonal rabbit anti-Aβ42 (Agrisera, Umeå, Sweden), and for measurements of soluble Aβ oligomers and protofibrils, plates were coated with 0.4 µg/mL of 82E1 (IBL International/Tecan Trading AG, Hamburg, Germany) overnight at 4 °C. Unspecific binding was blocked with 1% bovine serum albumin (BSA). For the soluble Aβx-42 measurements, 23 µL of TBS extract was added to 2.3 µL of 5% sodium dodecyl sulfate and heated for 5 min at 95 °C and further diluted 10 times. To study insoluble Aβx-42, formic acid extracts were neutralized with 2 M Tris and diluted 100 times. Additional TBS extracts were diluted 5 times for the study of Aβ protofibrils. The samples were loaded on plates and incubated overnight at 4 °C. Detection was carried out using biotinylated 6E10 (1 µg/mL; Nordic BioSite, Täby, Sweden) for Aβx-42, and biotinylated 82E1 (0.25 µg/mL; IBL International/Tecan Trading AG, Switzerland) for Aβ oligomers and protofibrils, followed by streptavidin-horse radish peroxidase (HRP; 1:4000; Mabtech AB, Nacka Strand, Sweden). Signals were developed with K blue aqueous TMB substrate, i.e. 3,3′,5,5′ tetramethylbenzidine and hydrogen peroxide (Neogen Corp., Lexington, KY, USA), and the reaction was stopped with 1 M H_2_SO_4_. Signals were read using a Tecan Infinite 200 pro plate reader (Tecan, Männedorf, Switzerland) at 450 nm. All brain samples were diluted in ELISA assay buffer (Mabtech AB, Nacka Strand, Sweden) to avoid interference from heterophilic antibodies^59^. Secondary antibodies and streptavidin-HRP were diluted in ELISA incubation buffer (PBS, 0.1% BSA, 0.05% Tween 20).

### Brain tissue binding assay

Regional BTB of the five model compounds was investigated in human post-mortem tissue. Available brain tissue material from 6 AD and 6 control donors, were allocated to BTB experiments as presented in Table S4. BTB was investigated in brain tissue homogenates using equilibrium dialysis according to previously published protocol with modifications^16^. For each compound, equilibrium dialysis was carried out during three sequential experimental days. Each daily experiment included brain regions from two randomly assigned control donors and two AD patients. A similar setup was used for all compounds. For the validation of inter-day variability, WB homogenate from one rat was included and analyzed in all dialysis experiments.

The BTB of the five compounds was also investigated in rats. FrCx, PrCx, BG, HIP, and CRB were collected from 24 rats and assembled in pools representative of two (paliperidone only) or three animals per pool, in total nine pools. Each drug was studied in 2–3 different pools representing biological replicates. Each experiment also included tissue homogenate from WB.

Independent of tissue origin, the brain tissue homogenates were prepared by diluting the tissue 1:9 (w:v) in 180 mM phosphate buffer saline (PBS), pH 7.4, and homogenizing the tissue using a VCX-130 ultrasonic processor (Sonics, Chemical Instruments AB, Sweden), at an amplitude of 40%. The homogenates were stored in aliquots at −80 °C. On the day of equilibrium dialysis, the homogenate aliquot was thawed and re-sonicated on ice before being spiked with the drug of interest, e.g., paliperidone, donepezil, memantine, indomethacin, or diazepam to a final concentration of 1 µM.

One day prior to the experiment, dialysis membrane strips with a molecular weight cut off of 12–14 kDa (HTDialysis LLC, Gales Ferry, CT, USA) were conditioned in PBS, pH 7.4, for 1 h and thereafter soaked overnight in 20% ethanol in PBS, pH 7.4. At the start of the experiment, the membranes were rinsed with PBS. A 96-well equilibrium dialysis apparatus (HTDialysis LLC, Gales Ferry, CT, USA) was assembled according to the manufacturer’s instructions. One hundred µL of PBS was added to one side of the dialysis membrane (hereafter referred to as buffer side). An equivalent volume of spiked brain homogenate was loaded in triplicates or duplicates to the opposite side of the membrane (hereafter referred to as tissue side). Incubation was carried out at 37 °C and at 200 rpm in a MaxQ 4450 benchtop shaker (Thermo Scientific, Waltham, MA, USA). To prevent pH changes and evaporation, adhesive sealing film was used to cover the samples.

After 6 h of incubation, 50 µL samples were transferred from both buffer and tissue sides to a Corning 0.5 mL, polypropylene round bottom 96-well plate (VWR, Stockholm, Sweden). To assure identical matrix composition for all samples, required for LC-MS/MS analysis of compounds, the buffer side samples were mixed with 50 µL of 1:9 (w:v) blank brain homogenate of the respective region in PBS and tissue side samples were mixed with 50 µL of PBS. The 96-well plate was sealed with aluminum film, vortexed and stored at −20 °C pending bioanalysis. To monitor relative recovery and stability of the compounds during equilibrium dialysis, samples were taken from the spiked brain homogenates at the start of experiment and after the 6 h incubation at 37 °C.

### Sample preparation for bioanalysis

Sample preparations were carried out on a Biomek4000 liquid handling system (Beckman Coulter, Bromma, Sweden) with Biomek Software version 3× . The sample preparation involved two key steps: i) protein precipitation by acetonitrile (1:3, v:v) and ii) dilution (1:4, v:v) of supernatant with mobile phase A (MPA, 0.1% formic acid in MilliQ water), described in detail below. Initially, samples, calibration standards, and blanks were thawed and vortexed for 5 min at 1200 rpm. Ninety µL of acetonitrile, containing 0.1–0.2% formic acid with 10–50 ng/mL of the respective internal standard was transferred into a polypropylene Corning 0.5 mL V-bottom 96-well plate (VWR, Stockholm, Sweden). The applied acetonitrile pipetting technique included pre-wetting, aspirate blowout and tip touch features and was carried out with an aspiration speed of 100 µL/s and a 7 µL air gap. Protein precipitation was achieved by adding 30 µL of sample into the acetonitrile containing wells. Sample transfer was performed using the default setting for “serum” pipetting, i.e., with pre-wetting, aspirate blowout, tip touch and an aspiration speed of 100 µL/s. Subsequently, the plate was vortexed for 5 minutes at 1200 rpm and centrifuged at 4100 rpm for 5 minutes in a Sigma 4–16KS (LABEX instrument AB, Helsingborg, Sweden). Using the default setting for “water” with an aspirate blowout feature, an 11 µL air gap and an aspiration speed of 100 µL/s, 150 µL of MPA was transferred to a polypropylene Corning 1 mL round bottom 96-well plate (VWR, Stockholm, Sweden). Fifty µL of supernatant was added to the MPA using an aspirate blowout feature with an aspiration speed of 100 µL/s. Plates were then vortexed and 5–10 µL was injected onto the column.

### Bioanalysis

Quantitative analysis of the compounds and their respective internal standards was achieved by liquid chromatography (LC) coupled to a multiple-reaction monitoring (MRM) tandem mass spectrometer (MS/MS), i.e., a Quatro Ultima^TM^ PT triple quadrupole mass spectrometer (Micromass, Waters, Manchester, United Kingdom). Reversed phase liquid chromatography was used for compound separation, utilizing either a HPLC or UPLC system. The HPLC system included a SIL-HTc autosampler (Schimadzu, Kyoto, Japan) and two LC-10ADvp pumps connected to a 50 × 4.6 mm HyPURITY 3 µm C18 column (Thermo scientific, USA), with a pre-column comprising the same material. The Acquity UPLC system consisted of a binary solvent manager and a sample organizer connected to an ACQUITY UPLC BEH C18 1.7 µm (2.1 × 50 mm) column (Waters Corporation, Tauton, Massachusetts, USA). Samples were eluted by a gradient program consisting of mobile phase A (MPA), containing 0.1% formic acid in MilliQ water, and of mobile phase B (MPB), containing 0.1% formic acid in 90:10 acetonitrile:MilliQ water. The gradient program had an initial concentration of 90% MPA and 10% MPB, which was ramped to an intermediate condition of 5% MPA and 95% MPB after 4 minutes.

MRM transition was carried out in positive electrospray (ES+) mode with capillary voltage 3 kV, source temperature 126 °C, desolvation temperature 450 °C, cone gas flow 50 L/h, and desolvation gas with a nitrogen flow of 1000 L/h. Argon was used as collision gas and the collision cell pressure was 3.13e-3 mbar. Mass transition details are presented in Table S6. Masslynx 4.1 (Micromass, Manchester, United Kingdom), together with the Application Manager QuanLynx was used for the quantification of drug concentrations.

A standard curve ranging from 1 nM to 1500 nM, including all five compounds, was prepared in 1:19 (w:v) rat WB homogenate in PBS to evaluate linearity of response and quantification of drugs in the rat samples. A second standard curve was prepared in 1:19 (w:v) human brain homogenate (mixture of all brain regions, including control subjects and AD patients) in PBS optimized based on prior information from rat BTB and ranging from 60 nM to 600 nM for human sample analysis. Standard curves weighted as 1/x were accepted at R^2^ values ≥ 0.99.

## Calculations

The fraction of unbound drug was first calculated in diluted brain homogenate for each region, f_u,hD,__brain,ROI_, as the buffer-to-tissue concentration ratio (Eq. 1). f_u,hD,brain,ROI_ was further used to calculate the fraction of unbound drug in brain regions, f_u,brain,ROI_ (Eq. 2), where the dilution factor, D, of the brain tissue homogenate was accounted for.

1 $${{\rm{f}}}_{{\rm{u}},{\rm{hD}},{\rm{brain}},{\rm{ROI}}}=\frac{{{\rm{C}}}_{{\rm{buffer}}}}{{{\rm{C}}}_{{\rm{tissue}}}}$$

2 $${{\rm{f}}}_{{\rm{u}},{\rm{brain}},{\rm{ROI}}}=\frac{\frac{1}{{\rm{D}}}}{((\frac{1}{{{\rm{f}}}_{{\rm{u}},{\rm{hD}},{\rm{brain}},{\rm{ROI}}}})-1)+\frac{1}{{\rm{D}}}}$$

A f_u,brain,ROI_ close to unity indicates a lack of nonspecific binding, while a value approaching zero indicates high nonspecific binding of the drug to cellular components and hence a low free fraction of the drug.

To ensure that compound concentrations were not affected by the equilibrium dialysis *per se*, sample stability as a % remaining drug during dialysis was evaluated by the collection of before and after incubation samples of spiked homogenates^34^. Stability was determined by the quantification of the drug in the respective homogenate after incubation for 6 h at 37 °C (C_hom,after_) versus the concentration before incubation (C_hom,before_), i.e. initial concentration, according to Equation 3.

3 $${\rm{Sample}}\,{\rm{stability}}\,( \% )=100\times \frac{{{\rm{C}}}_{{\rm{\hom }},{\rm{after}}}}{{{\rm{C}}}_{{\rm{\hom }},{\rm{before}}}}$$

### Statistical analysis

All analyses were performed using R version 3.3.1^60^ and the lme() function included in the nlme package^61^. Differences in the fraction of unbound drug between brain regions were assessed using linear mixed effects models. Brain region, pathology status (control or AD) and the interaction between them were entered as fixed effects with further adjustment for post-mortem interval in hours. Random intercepts for each individual as well as by-individual random slopes for brain regions were included in the model. Separate variances were allowed for the different brain regions as well as the different pathologies for both intercepts and slopes. f_u,brain,ROI_ values were log-transformed on the basis of visual inspection of plots of residuals versus fitted values which revealed clear heteroscedasticity when untransformed fraction values were used. For rats, the models included just a random intercept for each pool but the within region variances were allowed to differ. Detailed overview on LME models related to human brain regional tissue binding (represented in Fig. 3), rat brain regional tissue binding (represented in Fig. 5) and f_u,ROI_ as a function of AD markers (represented in Fig. 4) with their respective R-codes are presented in Supplementary information (See, Supplementary information, Code to reproduce the analyses).

Before analyzing the drugs separately, a test for any differences between AD and control groups was performed for all drugs simultaneously with the null hypothesis being that the difference in binding between AD and control are zero for all drugs and brain regions. Differences between brain regions were then tested, for each drug separately, by specifying a contrast matrix containing all differences of interest and performing a simultaneous test where the null hypothesis was that all contrasts are simultaneously equal to zero. Simultaneous 95% confidence intervals (CI) and multiplicity adjusted p-values were obtained using the multcomp package^62^ based on the joint multivariate normal distribution of the specific contrasts. This procedure takes the correlations between the test statistics into account and is less conservative and more powerful than the traditional Bonferroni method. The contrasts are differences in mean values on the log-scale, which were converted to ratios by anti-logging the contrasts and the corresponding confidence limits.

The associations between protein levels and the fraction of unbound drug were analyzed using linear mixed effects models. Replicate data on the individual level were averaged and models containing random intercepts for each individual were fitted with adjustment for brain region and post-mortem interval in hours. The 95% confidence intervals for the predicted fraction of unbound drug conditioned protein levels were obtained using a parametric bootstrap procedure in which 200 new sets of responses were simulated from the model with the random effects being generated from normal distributions with mean values and standard deviations (SD) based on those estimated from the models. The coefficient of determination (R^2^) was calculated according to Equation 30 in the paper by Nakagawa and Schielzeth, who define R^2^ as the fraction of the total variance that is explained by the variance of the fixed effects^63^.

## Supplementary information

Supplemantary information
