# Supplementary material for: Heterogeneous drug tissue binding in brain regions of rats, Alzheimer’s patients and controls: impact on translational drug development
Source: Sci Rep. 2019 Mar 29;9:5308. doi: 10.1038/s41598-019-41828-4 (PMC6440985; doi:10.1038/s41598-019-41828-4)
Supplement: Supplementary file 1 — Supplemantary information [file 41598_2019_41828_MOESM1_ESM.pdf]

Heterogeneous drug tissue binding in brain regions of rats, Alzheimer's patients and controls: impact on translational drug development

Sofia Gustafsson[1], Dag Sehlin[2], Erik Lampa[3], Margareta Hammarlund-Udenaes[1], Irena Loryan[1]*

[1]Translational PKPD Group, Department of Pharmaceutical Biosciences, Associate member of SciLifeLab, Uppsala University, Sweden.

[2] Molecular Geriatrics, Department of Public Health and Caring Sciences, Uppsala University, Sweden.

[3]Uppsala Clinical Research Center, Uppsala University, Sweden.

Table S1.

Table S1. Regional assessment of Aβ concentrations in human post-mortem brain tissue from AD (Group: AD) and control (Group: Control) donors, presented as minimum and maximum values. Aβ, amyloid beta; AD, Alzheimer's disease; FrCx, frontal cortex; PrCx, parietal cortex; BG, basal ganglia; CRB, cerebellum.

		AD		Control	
	**Region**	**Min (pg/mg brain)**	**Max (pg/mg brain)**	**Min (pg/mg brain)**	**Max (pg/mg brain)**
**Soluble Aβ x-42**	**FrCx**	46.92	96.17	0	20.94
	**PrCx**	51.83	78.90	0.32	26.25
	**BG**	70.00	78.79	0	0
	**CRB**	0.45	25.69	0	7.59
**Insoluble Aβ x-42**	**FrCx**	898.97	2951.52	0	3489.37
	**PrCx**	1096.40	2011.28	0	351.25
	**BG**	2578.65	3018.27	0	0
	**CRB**	0	1208.56	0	535.32
**Soluble oligomers and protofibrils**	**FrCx**	2.50	28.43	0.40	22.75
	**PrCx**	2.36	3.82	0.61	3.86
	**BG**	3.77	6.90	0.89	4.30
	**CRB**	1.35	12.30	0.90	6.06

Table S2.

Table S2. Between region comparison of drug brain tissue binding ($f_{u,brain,ROI}$), within and between groups; controls [C], and Alzheimer's disease patients [AD]. Data are presented as ratios of mean $f_{u,brain,ROI}$ with 95% simultaneous confidence interval lower and upper limits. FrCx, frontal cortex; PrCx, parietal cortex; BG, basal ganglia; CRB, cerebellum. p-values below 0.05 are presented in *italic*.

	**Diazepam**				**Donepezil**				**Indomethacin**				**Memantine**				**Paliperidone**			
**Comparison**	**Ratio**	**Lower**	**Upper**	**p-value**	**Ratio**	**Lower**	**Upper**	**p-value**	**Ratio**	**Lower**	**Upper**	**p-value**	**Ratio**	**Lower**	**Upper**	**p-value**	**Ratio**	**Lower**	**Upper**	**p-value**
**[C] PrCx - [C] FrCx**	0.943	0.799	1.113	0.959	0.930	0.768	1.127	0.939	0.893	0.683	1.167	0.893	0.939	0.780	1.131	0.968	0.960	0.816	1.129	0.994
**[C] BG - [C] FrCx**	0.871	0.714	1.063	0.400	0.900	0.720	1.126	0.830	0.819	0.595	1.129	0.537	0.967	0.777	1.203	1.000	0.917	0.764	1.101	0.831
**[C] CRB - [C] FrCx**	1.231	1.014	1.493	*0.027*	0.921	0.692	1.224	0.985	1.264	0.948	1.686	0.200	1.193	0.984	1.447	0.099	1.099	0.905	1.336	0.809
**[C] BG - [C] PrCx**	0.924	0.764	1.117	0.903	0.968	0.778	1.204	1.000	0.918	0.660	1.276	0.992	1.029	0.827	1.280	1.000	0.956	0.796	1.147	0.995
**[C] CRB - [C] PrCx**	1.305	1.085	1.568	*0.0004*	0.989	0.748	1.309	1.000	1.416	1.050	1.909	*0.011*	1.270	1.047	1.540	*0.005*	1.145	0.942	1.392	0.396
**[C] CRB - [C] BG**	1.412	1.140	1.750	*0.0001*	1.022	0.755	1.384	1.000	1.543	1.091	2.182	*0.004*	1.234	0.986	1.545	0.083	1.198	0.969	1.482	0.158
**[AD] PrCx - [AD] FrCx**	0.944	0.610	1.462	1.000	0.889	0.561	1.410	0.993	0.855	0.654	1.118	0.616	0.881	0.673	1.155	0.836	0.980	0.784	1.224	1.000
**[AD] BG - [AD] FrCx**	0.974	0.611	1.553	1.000	0.644	0.394	1.053	0.115	0.910	0.684	1.211	0.969	1.033	0.773	1.379	1.000	1.076	0.850	1.361	0.980
**[AD] CRB - [AD] FrCx**	1.210	0.750	1.954	0.923	0.548	0.314	0.956	*0.025*	1.365	0.975	1.911	0.092	1.303	0.989	1.717	0.069	1.490	1.156	1.921	*0.0001*
**[AD] BG - [AD] PrCx**	1.032	0.651	1.636	1.000	0.724	0.446	1.176	0.448	1.064	0.796	1.422	0.998	1.172	0.877	1.565	0.694	1.098	0.867	1.390	0.927
**[AD] CRB - [AD] PrCx**	1.282	0.798	2.058	0.741	0.616	0.355	1.069	0.128	1.596	1.135	2.244	*0.001*	1.479	1.123	1.948	*0.001*	1.520	1.179	1.961	*0.00003*
**[AD] CRB - [AD] BG**	1.243	0.752	2.053	0.886	0.851	0.478	1.515	0.988	1.500	1.051	2.141	*0.014*	1.262	0.940	1.694	0.233	1.385	1.060	1.810	*0.006*
**[AD] FrCx - [C] FrCx**	1.185	0.785	1.789	0.910	1.492	1.019	2.184	*0.033*	1.063	0.799	1.415	0.998	1.153	0.880	1.512	0.733	1.154	0.868	1.535	0.782
**[AD] PrCx - [C] PrCx**	1.186	0.794	1.773	0.894	1.426	0.986	2.063	0.069	1.018	0.752	1.378	1.000	1.082	0.826	1.418	0.985	1.178	0.885	1.568	0.647
**[AD] BG - [C] BG**	1.325	0.850	2.066	0.520	1.068	0.701	1.627	1.000	1.180	0.821	1.697	0.848	1.232	0.903	1.681	0.436	1.353	0.996	1.838	0.057
**[AD] CRB - [C] CRB**	1.165	0.733	1.853	0.971	0.888	0.524	1.507	0.997	1.148	0.782	1.685	0.953	1.260	0.952	1.668	0.189	1.564	1.122	2.180	*0.002*

Table S3.

Table S3. Between region comparison of drug brain tissue binding ($f_{u,brain,ROI}$) in healthy Sprague-Dawley. Data are presented as ratios of mean $f_{u,brain,ROI}$ with 95% simultaneous confidence interval lower and upper limits. FrCx, frontal cortex; PrCx, parietal cortex; BG, basal ganglia; CRB, cerebellum; HIP, hippocampus; WB, whole brain. p-values below 0.05 presented in *italic*.

	**Diazepam**				**Donepezil**				**Indomethacin**				**Memantine**				**Paliperidone***			
**Comparison**	**Ratio**	**Lower**	**Upper**	**p-value**	**Ratio**	**Lower**	**Upper**	**p-value**	**Ratio**	**Lower**	**Upper**	**p-value**	**Ratio**	**Lower**	**Upper**	**p-value**	**Ratio**	**Lower**	**Upper**	**p-value**
**[R] PrCx - [R] FrCx**	1.191	0.976	1.453	0.120	0.988	0.821	1.189	1.000	0.997	0.817	1.216	1.000	0.948	0.713	1.262	0.995	na	na	na	na
**[R] BG - [R] FrCx**	1.227	0.988	1.523	0.076	1.003	0.828	1.216	1.000	1.003	0.810	1.243	1.000	1.030	0.817	1.300	0.999	0.940	0.647	1.367	0.991
**[R] CRB - [R] FrCx**	1.545	1.298	1.838	*<0.001*	0.794	0.638	0.988	*0.031*	1.068	0.818	1.395	0.981	0.958	0.746	1.231	0.997	1.030	0.721	1.473	0.999
**[R] HIP - [R] FrCx**	1.323	1.080	1.620	*<0.001*	1.108	0.916	1.341	0.640	1.039	0.847	1.274	0.995	1.142	0.895	1.456	0.625	1.092	0.765	1.558	0.962
**[R] WB - [R] FrCx**	1.169	0.964	1.416	0.186	0.975	0.815	1.166	0.999	0.978	0.788	1.214	1.000	0.956	0.714	1.278	0.998	1.016	0.719	1.435	1.000
**[R] BG - [R] PrCx**	1.030	0.862	1.231	0.997	1.015	0.876	1.176	1.000	1.006	0.880	1.150	1.000	1.087	0.870	1.357	0.893	na	na	na	na
**[R] CRB - [R] PrCx**	1.297	1.146	1.469	*<0.001*	0.803	0.671	0.962	*0.007*	1.072	0.870	1.319	0.932	1.010	0.794	1.286	1.000	na	na	na	na
**[R] HIP - [R] PrCx**	1.111	0.945	1.306	0.427	1.121	0.970	1.296	0.214	1.042	0.927	1.171	0.916	1.204	0.953	1.520	0.207	na	na	na	na
**[R] WB - [R] PrCx**	0.981	0.847	1.137	0.999	0.987	0.868	1.122	1.000	0.981	0.855	1.125	0.999	1.008	0.759	1.337	1.000	na	na	na	na
**[R] CRB - [R] BG**	1.260	1.084	1.464	*<0.001*	0.791	0.656	0.953	*0.005*	1.065	0.852	1.331	0.965	0.930	0.781	1.107	0.841	1.096	0.786	1.528	0.944
**[R] HIP - [R] BG**	1.078	0.898	1.295	0.843	1.104	0.947	1.288	0.437	1.036	0.898	1.194	0.981	1.108	0.940	1.305	0.474	1.161	0.834	1.617	0.731
**[R] WB - [R] BG**	0.953	0.805	1.127	0.963	0.972	0.845	1.117	0.992	0.975	0.832	1.143	0.998	0.927	0.737	1.166	0.936	1.081	0.785	1.487	0.964
**[R] HIP - [R] CRB**	0.856	0.752	0.976	*0.010*	1.396	1.163	1.675	*<0.001*	0.972	0.785	1.203	0.999	1.191	0.986	1.439	0.087	1.060	0.776	1.447	0.987
**[R] WB - [R] CRB**	0.757	0.677	0.845	*<0.001*	1.228	1.041	1.449	*0.005*	0.915	0.731	1.146	0.870	0.997	0.778	1.277	1.000	0.986	0.731	1.331	1.000
**[R] WB - [R] HIP**	0.883	0.759	1.029	0.182	0.880	0.770	1.005	0.068	0.942	0.814	1.089	0.842	0.837	0.658	1.064	0.280	0.931	0.691	1.253	0.965

*Rat $f_{u,brain,ROI}$ values for paliperidone have been taken from a previous study by Loryan et al. [1]

Table S4.

Table S4. Post-mortem human tissue availability for the brain tissue binding and Aβ individual measurements. Aβ, amyloid beta; AD, Alzheimer's disease; FrCx, frontal cortex; PrCx, parietal cortex; BG, basal ganglia; CRB, cerebellum; na, not available.

		Available brain regions for brain tissue binding assessment				Available brain regions for pathological assessment of Aβ			
	**ID no.**	**FrCx**	**PrCx**	**BG**	**CRB**	**FrCx**	**PrCx**	**BG**	**CRB**
**Control (n=6)**	ID 1	x	x	x	x	x	x	x	x
	ID 2	x	x	x	x	x	x	na	x
	ID 3	x	x	na	x	x	x	na	x
	ID 4	x	x	x	x	x	x	x	x
	ID 5	x	x	na	x	x	x	na	x
	ID 6	x	x/na*	x/na*	x/na*	x	x	x	x
**AD (n=6)**	ID 7	x	x	x	x	x	x	x	x
	ID 8	x	x	x	x	na	na	na	x
	ID 9	x	x	na	x	x	x	na	x
	ID 10	x	x	x	x	x	x	na	x
	ID 11	x	x	x	x	x	x	na	x
	ID 12	x	x	x	x/na**	x	na	x	x

*Not available for indomethacin $f_{u,brain}$ measurements

**Not available for diazepam, indomethacin, and paliperidone $f_{u,brain}$ measurements.

Table S5.

Table S5. Molecular weight, ion class, cLogP and predicted pKa for diazepam, donepezil, indomethacin, memantine and paliperidone

Drug	Molecular Weight	Ion Class	clogP	ACDpKa (Base/acid)
**Diazepam**	284.75	Neutral	2.96	3.4
**Donepezil**	379.49	Base	4.2	8.6
**Indomethacin**	357.79	Acid	4.18	4
**Memantine**	179.3	Base	3.3	10.7
**Paliperidone**	426.5	Base	2.4	8.2/2.6

Table S6.

Table S6. MRM transition, collision energy (eV), and cone voltage for diazepam, donepezil, indomethacin, memantine and paliperidone with their respective internal standards.

Compound	MRM transition	Collision energy (eV)	Cone voltage (V)
**Diazepam**	284.9>153.8	24	100
**Diazepam-D5**	289.9>153.8	24	100
**Donepezil**	380.10 > 91.20	30	40
**Donepezil-D5**	385.2 > 96.00	30	40
**Indomethacin**	358.0 >138.8	19	35
**Indomethacin-D7**	362.0 >143.0	19	35
**Memantine**	180.1 > 162.9	14	40
**Memantine-D6**	186.0 > 168.9	14	40
**Paliperidone**	427.0 >206.9	26	130
**Paliperidone–D4**	431.0 >210.9	26	130

Fig. S1.

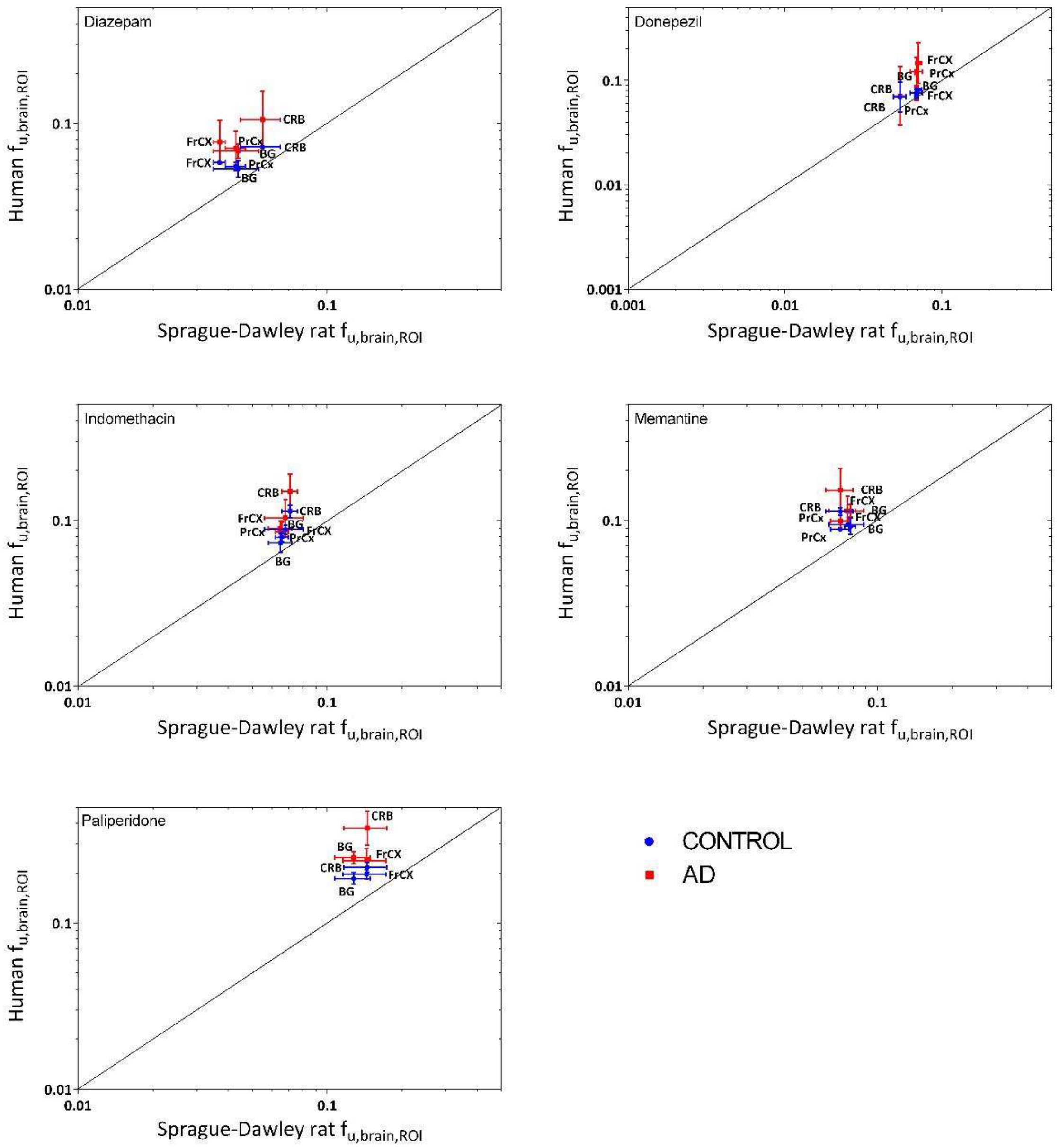

Fig. S1. Relationship between fraction of unbound drug in brain ROIs ($f_{u,brain,ROI}$) in healthy Sprague-Dawley rats and humans including controls (blue circles) and AD patients (red squares). The line of identity is indicated as a solid line. AD, Alzheimer's disease; FrCx, frontal cortex; PrCx, parietal cortex; BG, basal ganglia; CRB, cerebellum.

References

1 Loryan, I. *et al.* In-depth neuropharmacokinetic analysis of antipsychotics based on a novel approach to estimate unbound target-site concentration in CNS regions: link to spatial receptor occupancy. *Mol Psychiatry* **21**, 1527-1536, doi:10.1038/mp.2015.229 (2016).

Code to reproduce the analyses

March 8, 2019

1 Introduction

This document describes the R-code used for the analyses in the paper. First, we load the necessary packages.

```
library("lattice")
library("contrast")
library("nlme")
library("multcomp")
```

2 Human brain tissue binding

Assume that the data are stored in a list with the drugs as elements.

The models are then fitted with

```
fits <- lapply(d, function(x) {
    lme(log(fu.rois) ~ pathology.short +
            rois + pathology.short:rois +
            postmortem.time.h,
        random = list(id = ~1, rois = pdDiag(~pathology.short)),
        weights = varComb(varIdent(form = ~1|rois),
                          varIdent(form = ~1|pathology.short)),
        data = x,
        subset = pathology.short %in% c("Control", "AD"))
})
```

where we allow for different variance components for the different brain regions and diseases. The coefficients themselves are not of much interest but we present them for completeness sake.

```
betas
```

```
## $Diazepam
##                            Value Std.Error DF t-value p-value
## (Intercept)                -3.04      0.12 83  -26.35    0.00
## pathology.shortAD           0.17      0.14  9    1.19    0.26
## roisPrCx                   -0.06      0.06 26   -1.02    0.32
## roisBG                     -0.14      0.07 26   -2.00    0.06
## roisCRB                     0.21      0.07 26    3.10    0.00
## postmortem.time.h           0.01      0.00  9    1.91    0.09
## pathology.shortAD:roisPrCx  0.00      0.16 26    0.01    0.99
## pathology.shortAD:roisBG    0.11      0.18 26    0.64    0.53
## pathology.shortAD:roisCRB  -0.02      0.18 26   -0.09    0.93
##
## $Donepezil
##                            Value Std.Error DF t-value p-value
## (Intercept)                -2.75      0.09 78  -30.65    0.00
## pathology.shortAD           0.40      0.13  9    3.03    0.01
## roisPrCx                   -0.07      0.07 27   -1.09    0.29
## roisBG                     -0.10      0.08 27   -1.35    0.19
## roisCRB                    -0.08      0.10 27   -0.84    0.41
## postmortem.time.h           0.01      0.00  9    3.15    0.01
## pathology.shortAD:roisPrCx -0.05      0.17 27   -0.26    0.80
## pathology.shortAD:roisBG   -0.33      0.19 27   -1.79    0.09
## pathology.shortAD:roisCRB  -0.52      0.22 27   -2.39    0.02
##
## $Indomethacin
##                            Value Std.Error DF t-value p-value
## (Intercept)                -2.59      0.09 77  -29.38    0.00
## pathology.shortAD           0.06      0.10  9    0.62    0.55
## roisPrCx                   -0.11      0.09 23   -1.22    0.23
## roisBG                     -0.20      0.11 23   -1.80    0.09
## roisCRB                     0.23      0.10 23    2.35    0.03
## postmortem.time.h           0.01      0.00  9    2.63    0.03
## pathology.shortAD:roisPrCx -0.04      0.13 23   -0.33    0.74
## pathology.shortAD:roisBG    0.10      0.15 23    0.71    0.49
## pathology.shortAD:roisCRB   0.08      0.15 23    0.50    0.62
##
## $Memantine
##                            Value Std.Error DF t-value p-value
```

```
## (Intercept)                 -2.47      0.08 84  -31.43    0.00
## pathology.shortAD            0.14      0.09  9    1.53    0.16
## roisPrCx                    -0.06      0.06 27   -0.97    0.34
## roisBG                      -0.03      0.08 27   -0.45    0.66
## roisCRB                      0.18      0.07 27    2.65    0.01
## postmortem.time.h            0.01      0.00  9    1.79    0.11
## pathology.shortAD:roisPrCx -0.06      0.11 27   -0.56    0.58
## pathology.shortAD:roisBG    0.07      0.13 27    0.53    0.60
## pathology.shortAD:roisCRB   0.09      0.12 27    0.76    0.45
##
## $Paliperidone
##                             Value Std.Error DF t-value p-value
## (Intercept)                 -1.75      0.09 81  -18.58    0.00
## pathology.shortAD            0.14      0.10  9    1.45    0.18
## roisPrCx                    -0.04      0.06 26   -0.73    0.47
## roisBG                      -0.09      0.06 26   -1.37    0.18
## roisCRB                      0.09      0.07 26    1.41    0.17
## postmortem.time.h            0.01      0.00  9    1.52    0.16
## pathology.shortAD:roisPrCx  0.02      0.10 26    0.22    0.83
## pathology.shortAD:roisBG    0.16      0.10 26    1.54    0.13
## pathology.shortAD:roisCRB   0.30      0.11 26    2.75    0.01
```

Set up a contrast matrix corresponding to the different comparisons of interest. Use the first model to generate the contrast matrix, the only difference between the models are the outcomes. The postmortem time was set to 14h which was the median value in the data.

```
## Fit model to the first data set
fit <- lme(log(fu.rois) ~ pathology.short + rois +
               pathology.short:rois +
               postmortem.time.h,
           random = list(id = ~1, rois = pdDiag(~pathology.short)),
           weights = varComb(varIdent(form = ~1|rois),
                             varIdent(form = ~1|pathology.short)),
           data = d[[1]],
           subset = pathology.short %in% c("Control", "AD"))

## First pairwise comparisons with FrCx within Control
K1 <- contrast(fit,
```

```r
                  a = list(pathology.short = "Control",
                           rois = c("PrCx", "BG", "CRB"),
                           postmortem.time.h = 14),
                  b = list(pathology.short = "Control",
                           rois = "FrCx",
                           postmortem.time.h = 14))$X

## Then comparisons with PrCx. Don't need to include
## FrCx since contrast already tested
K2 <- contrast(fit,
                  a = list(pathology.short = "Control",
                           rois = c("BG", "CRB"),
                           postmortem.time.h = 14),
                  b = list(pathology.short = "Control",
                           rois = "PrCx",
                           postmortem.time.h = 14))$X

## And the last comparison
K3 <- contrast(fit,
                  a = list(pathology.short = "Control",
                           rois = "CRB",
                           postmortem.time.h = 14),
                  b = list(pathology.short = "Control", rois = "BG",
                           postmortem.time.h = 14))$X

## Now the same for the comparisons within AD
K4 <- contrast(fit,
                  a = list(pathology.short = "AD",
                           rois = c("PrCx", "BG", "CRB"),
                           postmortem.time.h = 14),
                  b = list(pathology.short = "AD",
                           rois = "FrCx",
                           postmortem.time.h = 14))$X
K5 <- contrast(fit,
                  a = list(pathology.short = "AD",
                           rois = c("BG", "CRB"),
                           postmortem.time.h = 14),
                  b = list(pathology.short = "AD",
                           rois = "PrCx",
```

```
                        postmortem.time.h = 14))$X
K6 <- contrast(fit,
               a = list(pathology.short = "AD",
                        rois = "CRB",
                        postmortem.time.h = 14),
               b = list(pathology.short = "AD",
                        rois = "BG",
                        postmortem.time.h = 14))$X

## And comparisons between AD and Control
K7 <- contrast(fit,
               a = list(pathology.short = "AD",
                        rois = "FrCx",
                        postmortem.time.h = 14),
               b = list(pathology.short = "Control",
                        rois = "FrCx",
                        postmortem.time.h = 14))$X
K8 <- contrast(fit,
               a = list(pathology.short = "AD",
                        rois = "PrCx",
                        postmortem.time.h = 14),
               b = list(pathology.short = "Control",
                        rois = "PrCx",
                        postmortem.time.h = 14))$X
K9 <- contrast(fit,
               a = list(pathology.short = "AD",
                        rois = "BG",
                        postmortem.time.h = 14),
               b = list(pathology.short = "Control",
                        rois = "BG",
                        postmortem.time.h = 14))$X
K10 <- contrast(fit,
                a = list(pathology.short = "AD",
                         rois = "CRB",
                         postmortem.time.h = 14),
                b = list(pathology.short = "Control",
                         rois = "CRB",
                         postmortem.time.h = 14))$X
```

```
K <- rbind(K1, K2, K3, K4, K5, K6, K7, K8, K9, K10)

rownames(K) <- c("[C] PrCx - [C] FrCx",
                 "[C] BG - [C] FrCx",
                 "[C] CRB - [C] FrCx",
                 "[C] BG - [C] PrCx",
                 "[C] CRB - [C] PrCx",
                 "[C] CRB - [C] BG",
                 "[AD] PrCx - [AD] FrCx",
                 "[AD] BG - [AD] FrCx",
                 "[AD] CRB - [AD] FrCx",
                 "[AD] BG - [AD] PrCx",
                 "[AD] CRB - [AD] PrCx",
                 "[AD] CRB - [AD] BG",
                 "[AD] FrCx - [C] FrCx",
                 "[AD] PrCx - [C] PrCx",
                 "[AD] BG - [C] BG",
                 "[AD] CRB - [C] CRB")
```

With the contrast matrix in place, we use the `glht()` function to estimate the contrasts and get simultaneous confidence intervals. The code below reproduces figure 3 in the paper.

```
g <- lapply(fits, function(x) glht(x, linfct = K))
cis <- lapply(g, function(x) {
                  ci <- confint(x)$confint
                  cid <- as.data.frame(ci)
                  cid$comp <- rownames(ci)
                  cid
              })

names(cis) <- drugs
cis <- do.call("rbind", cis)
cis$drug <- sapply(rownames(cis), function(x) {
    strsplit(x, ".", fixed = TRUE)
}[[1]][1])

cis$comp <- factor(cis$comp)
cis$comp <- factor(cis$comp,
                   levels = levels(cis$comp)[
```

```
                       rev(match(rownames(K), levels(cis$comp)))])

xplot(comp ~ exp(Estimate)|drug, data = cis, layout = c(5, 1),
       panel = function(x, y, subscripts, ...) {
           panel.abline(v = 0, lty = 3)
           panel.abline(h = c(4.5, 10.5), lty = 1)
           panel.segments(x0 = cis$lwr[subscripts],
                          y0 = y,
                          x1 = cis$upr[subscripts],
                          y1 = y, col = "black")
           panel.xyplot(x, y, pch = 16, col = "black", ...)
       }, xlim = c(exp(min(cis$lwr) - 0.1),
                   exp(max(cis$upr) + 0.1)),
       xlab = "Ratio", ylab = "",
       scales = list(x = list(log = "e",
                              at = seq(0.5, 2.5, by = 0.5),
                              alternating = FALSE,
                              tck = c(1, 0))),
       strip = strip.custom(bg = "grey95"))
```

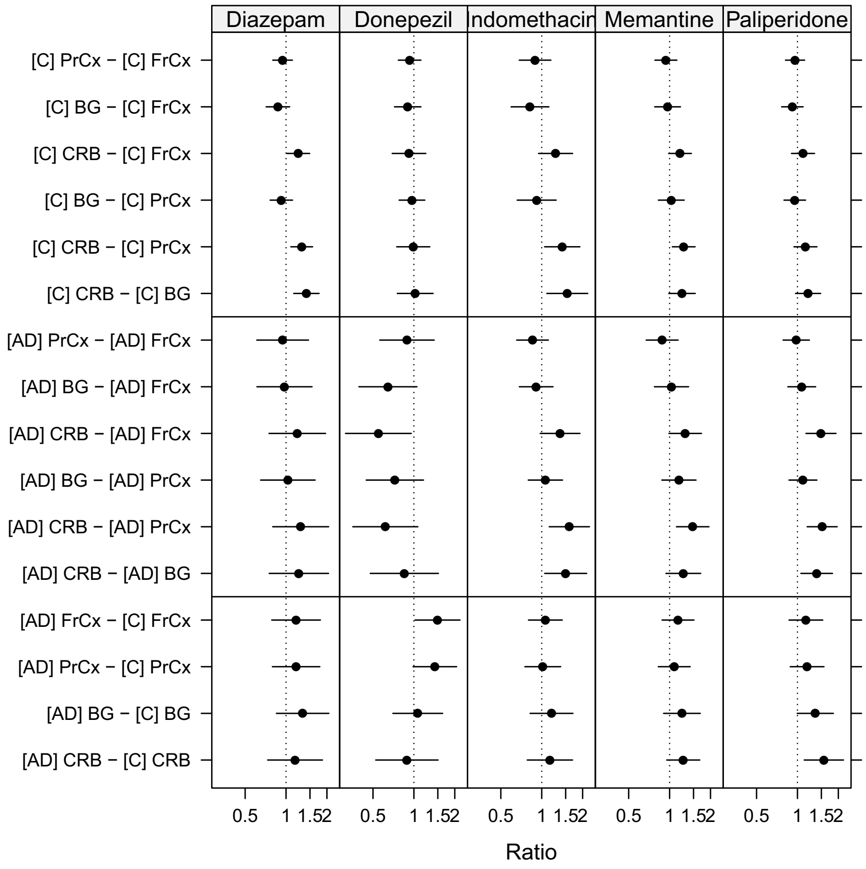

3 Rat brain tissue binding

A similar approach is done with the rat data. The models are a bit simpler as we do not have repeated measurements on the individual rats.

```
fits.rat <- lapply(d, function(x) {
                   lme(log(fu.rois) ~ rois,
                       random = list(id = ~1),
                       weights = varIdent(form = ~1|rois), data = x,
                       subset = pathology.short == "Rat")
})
```

With coefficients

```
betas.rat

## $Diazepam
##             Value Std.Error DF t-value p-value
## (Intercept) -3.34      0.10 45  -32.39    0.00
## roisPrCx     0.17      0.07 45    2.49    0.02
## roisBG       0.20      0.08 45    2.67    0.01
## roisCRB      0.44      0.06 45    7.07    0.00
## roisHIP      0.28      0.07 45    3.90    0.00
## roisWB       0.16      0.07 45    2.29    0.03
##
## $Donepezil
##             Value Std.Error DF t-value p-value
## (Intercept) -2.71      0.08 33  -33.53    0.00
## roisPrCx    -0.01      0.07 33   -0.18    0.86
## roisBG       0.00      0.07 33    0.05    0.96
## roisCRB     -0.23      0.08 33   -3.00    0.01
## roisHIP      0.10      0.07 33    1.52    0.14
## roisWB      -0.03      0.06 33   -0.40    0.69
##
## $Indomethacin
##             Value Std.Error DF t-value p-value
## (Intercept) -2.73      0.07 44  -41.89    0.00
## roisPrCx     0.00      0.07 44   -0.04    0.97
## roisBG       0.00      0.08 44    0.04    0.97
## roisCRB      0.07      0.09 44    0.70    0.49
## roisHIP      0.04      0.07 44    0.52    0.60
## roisWB      -0.02      0.08 44   -0.29    0.77
##
## $Memantine
##             Value Std.Error DF t-value p-value
## (Intercept) -2.59      0.07 31  -35.32    0.00
## roisPrCx    -0.05      0.10 31   -0.53    0.60
## roisBG       0.03      0.08 31    0.37    0.72
## roisCRB     -0.04      0.09 31   -0.48    0.63
## roisHIP      0.13      0.09 31    1.54    0.13
## roisWB      -0.05      0.10 31   -0.44    0.66
##
```

```
## $Paliperidone
##               Value Std.Error DF  t-value p-value
## (Intercept) -1.99      0.10 25  -19.33    0.00
## roisBG      -0.06      0.14 25   -0.45    0.66
## roisCRB      0.03      0.13 25    0.23    0.82
## roisHIP      0.09      0.13 25    0.67    0.51
## roisWB       0.02      0.13 25    0.12    0.90
```

Generate the contrast matrix, again using the first model to generate the contrasts.

```
fit.r <- lme(log(fu.rois) ~ rois,
                      random = list(id = ~1),
                      weights = varIdent(form = ~1|rois), data = d[[1]],
                      subset = pathology.short == "Rat")

K1r <- contrast(fit.r,
               a = list(rois = c("PrCx", "BG", "CRB", "HIP", "WB")),
               b = list(rois = "FrCx"))$X
K2r <- contrast(fit.r,
               a = list(rois = c("BG", "CRB", "HIP", "WB")),
               b = list(rois = "PrCx"))$X
K3r <- contrast(fit.r,
               a = list(rois = c("CRB", "HIP", "WB")),
               b = list(rois = "BG"))$X
K4r <- contrast(fit.r,
               a = list(rois = c("HIP", "WB")),
               b = list(rois = "CRB"))$X
K5r <- contrast(fit.r,
               a = list(rois = "WB"),
               b = list(rois = "HIP"))$X
Kr <- rbind(K1r, K2r, K3r, K4r, K5r)

rownames(Kr) <- c("[R] PrCx - [R] FrCx",
                  "[R] BG - [R] FrCx",
                  "[R] CRB - [R] FrCx",
                  "[R] HIP - [R] FrCx",
                  "[R] WB - [R] FrCx",
                  "[R] BG - [R] PrCx",
                  "[R] CRB - [R] PrCx",
```

```
                  "[R] HIP - [R] PrCx",
                  "[R] WB - [R] PrCx",
                  "[R] CRB - [R] BG",
                  "[R] HIP - [R] BG",
                  "[R] WB - [R] BG",
                  "[R] HIP - [R] CRB",
                  "[R] WB - [R] CRB",
                  "[R] WB - [R] HIP")
```

And estimate the contrasts as well as the simultaneous confidence intervals. The figure corresponds to Figure 5 in the paper.

```
gr <- lapply(fits.rat,
             function(x) {
                 if (length(fixef(x)) == 6) {
                     glht(x, linfct = Kr)
                 } else {
                     glht(x,
                          linfct =
                              Kr[-grep("PrCx",rownames(Kr)),
                                 -grep("PrCx", colnames(Kr))])
               }})

cisr <- lapply(gr, function(x) {
                  ci <- confint(x)$confint
                  cid <- as.data.frame(ci)
                  cid$comp <- rownames(ci)
                  cid
              })

psr <- lapply(gr, function(x) {
    su <- summary(x)
    ps <- su$test$pvalues
    da <- data.frame(p = ps,comp = names(su$test$coefficients))
    da
})

names(cisr) <- drugs
cisr <- do.call("rbind", cisr)
cisr$drug <- sapply(
```

```
    rownames(cisr),
    function(x) {
        strsplit(x, ".", fixed = TRUE)
    }[[1]][1])

cisr$comp <- factor(cisr$comp)
cisr$comp <- factor(cisr$comp,
                    levels = levels(cisr$comp)[
                        rev(match(rownames(Kr),
                                  levels(cisr$comp)))])

names(psr) <- drugs
psr <- do.call("rbind", psr)
psr$drug <- sapply(
    rownames(psr), function(x) {
        strsplit(x, ".", fixed = TRUE)
    }[[1]][1])

psr$comp <- factor(psr$comp)
psr$comp <- factor(psr$comp,
                   levels = levels(psr$comp)[
                       rev(match(rownames(Kr),
                                 levels(psr$comp)))])



xyplot(comp ~ exp(Estimate)|drug,
       data = cisr, layout = c(5, 1),
       panel = function(x, y, subscripts, ...) {
           panel.abline(v = 0, lty = 3)
           panel.segments(x0 = cisr$lwr[subscripts],
                          y0 = y,
                          x1 = cisr$upr[subscripts],
                          y1 = y, col = "black")
           panel.xyplot(x, y,
                        pch = 16, col = "black", ...)
       }, xlim = c(exp(min(cisr$lwr) - 0.5),
                   exp(max(cisr$upr) + 0.5)),
       xlab = "Ratio", ylab = "",
```

```
        scales = list(x = list(log = "e",
                               at = c(0.5, 1, 1.5, 2.5),
                               alternating = FALSE,
                               tck = c(1, 0))),
        strip = strip.custom(bg = "grey95"))
```

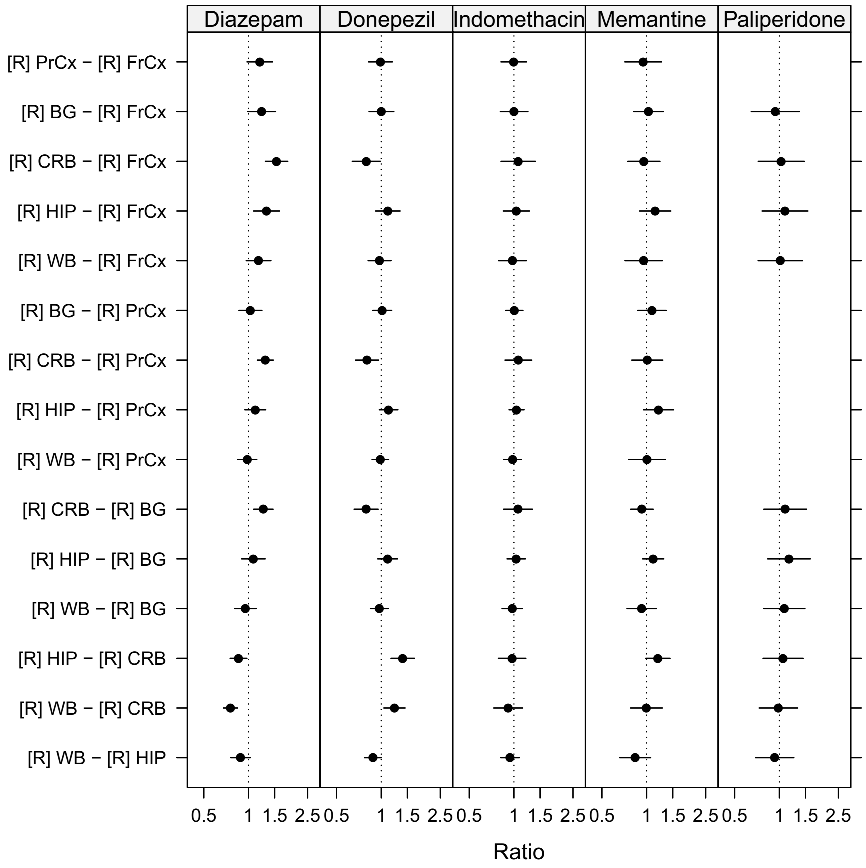

4 Human brain tissue binding vs AD markers

We average the $f_{u,ROI}$ values by individual since the AD marker levels are measured per individual instead of per brain region and stack the data into a `data.frame`.

```r
library("lme4")
library("piecewiseSEM")
library("grid")

## Fit the models

ff <- lmer(lfu ~ drug*(I(tbsabetax42pgmgbrain)) +
               (1|id) + (1|rois),
           data = d, na.action = na.omit)

## Divide by 1,000 for numerical stability
ff2 <- lmer(lfu ~ drug * (I(faabetax42pgmgbrain/1000)) +
                (1|rois) + (1|id),
            data = d, na.action = na.omit)


## Re-fit the models one drug at a time to
## pass the resulting list to rsquared()
ff1list <- lapply(levels(d$drug), function(x) {
    fit <- lmer(lfu ~ tbsabetax42pgmgbrain +
                    (1|rois) + (1|id),
                data = d,
                subset = drug == x,
                na.action = na.omit)
  fit
})


ff2list <- lapply(levels(d$drug), function(x) {
    fit <- lmer(lfu ~ I(faabetax42pgmgbrain/1000) +
                    (1|rois) + (1|id),
                data = d,
                subset = drug == x,
                na.action = na.omit)
    fit
})

r2 <- rsquared(ff1list)[,5]
```

```r
r22 <- rsquared(ff2list)[,5]

newX <- expand.grid(
    drug = unique(d$drug),
    tbsabetax42pgmgbrain = seq(0, 90, length = 100))

newX2 <- expand.grid(
    drug = unique(d$drug),
    faabetax42pgmgbrain = seq(0, 2500, length = 100))

set.seed(1)
res <- bootMer(x = ff,
               FUN = function(x) {
                   predict(x, newdata = newX, re.form = NA)
               }, nsim = 1000)
lims <- t(apply(res$t, 2, quantile, c(0.025, 0.975)))
newX$pred <- plogis(
    predict(ff, newdata = newX,
            re.form = NA))

newX$lower <- plogis(lims[,1])
newX$upper <- plogis(lims[,2])

set.seed(1)
res2 <- bootMer(x = ff2,
                FUN = function(x) {
                    predict(x, newdata = newX2, re.form = NA)
                }, nsim = 1000)
lims2 <- t(apply(res2$t, 2, quantile, c(0.025, 0.975)))
newX2$pred <- plogis(
    predict(ff2, newdata = newX2,
            re.form = NA))

newX2$lower <- plogis(lims2[,1])
newX2$upper <- plogis(lims2[,2])
```

```r
xyplot(pred ~ faabetax42pgmgbrain|drug,
       data = newX2, ylim = c(-0.02, 0.37),
```

```
    panel = function(x, y, subscripts, ...) {
        panel.xyplot(x, y, type = "l",
                     col = "black",
                     lwd = 2)
        panel.lines(x = x,
                    y = newX2$lower[subscripts],
                    col = "black")
        panel.lines(x = x,
                    y = newX2$upper[subscripts],
                    col = "black")
        r2 <- sprintf("%1.2f", r22[panel.number()])
        grid.text(if(r2 >= 0.01) bquote(R^2 == .(r2))
                  else expression(R^2 < 0.01),
                  x = unit(0, "native"),
                  y = unit(0, "native"),
                  hjust = 0, gp = gpar(cex = 0.75))
    }, layout = c(5, 1),
    ylab = "Fraction of unbound drug in brain regions",
    xlab = expression(
        paste("Insoluble A", beta, "x - 42 (pg/mg brain)")
    ), scales = list(
           y = list(at = seq(0, 0.45, by = 0.05))),
    strip = strip.custom(bg = "grey95"))
```

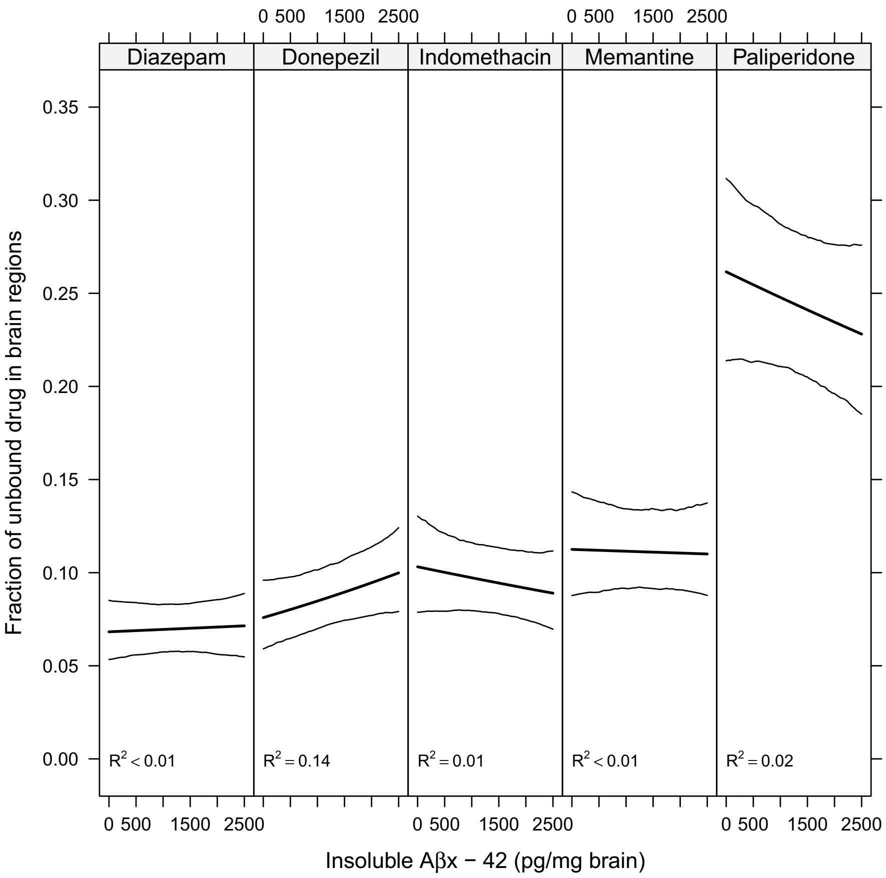


```
xyplot(pred ~ tbsabetax42pgmgbrain|drug,
       data = newX, ylim = c(-0.02, 0.37),
       panel = function(x, y, subscripts, ...) {
           panel.xyplot(x, y, type = "l",
                        col = "black",
                        lwd = 2)
           panel.lines(x = x,
                       y = newX$lower[subscripts],
                       col = "black")
           panel.lines(x = x,
                       y = newX$upper[subscripts],
                       col = "black")
```

```r
        r2 <- sprintf("%1.2f", r2[panel.number()])
        grid.text(if(r2 >= 0.01) bquote(R^2 == .(r2))
                  else expression(R^2 < 0.01),
                  x = unit(0, "native"),
                  y = unit(0, "native"),
                  hjust = 0, gp = gpar(cex = 0.75))
    }, layout = c(5, 1),
    ylab = "Fraction of unbound drug in brain regions",
    xlab = expression(
        paste("Soluble A", beta, "x - 42 (pg/mg brain)")
    ), scales = list(
           y = list(at = seq(0, 0.45, by = 0.05))),
    strip = strip.custom(bg = "grey95"))
```

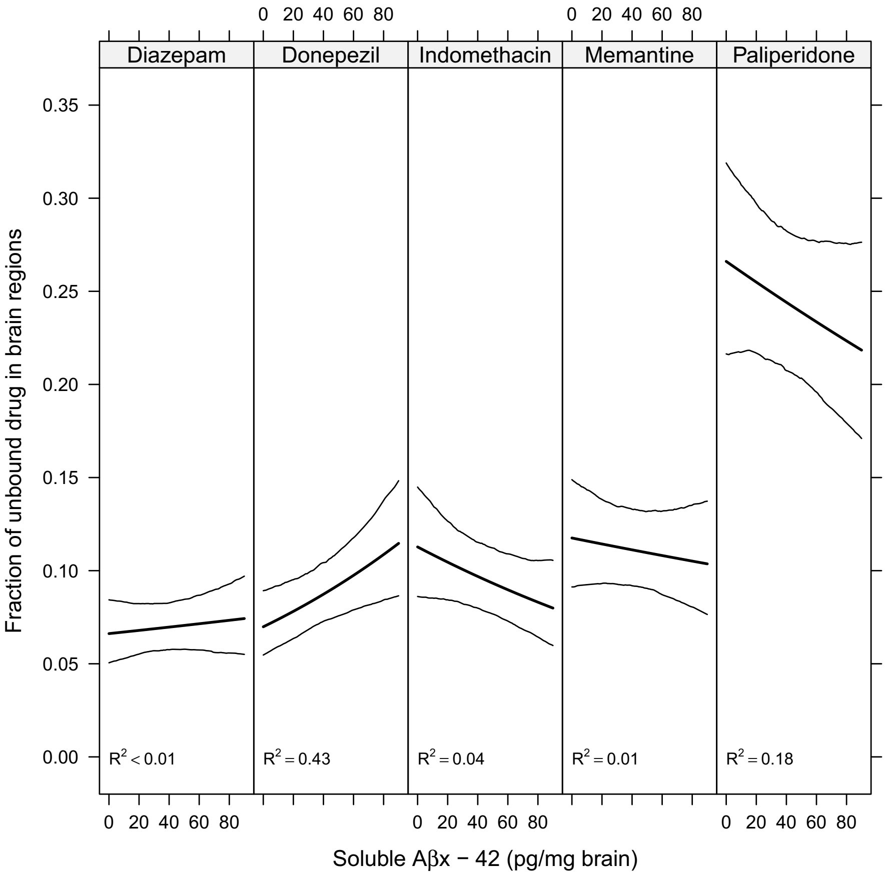

5 R session info

```
sessionInfo()

## R version 3.5.0 (2018-04-23)
## Platform: x86_64-w64-mingw32/x64 (64-bit)
## Running under: Windows 7 x64 (build 7601) Service Pack 1
##
## Matrix products: default
##
## locale:
```

```
## [1] LC_COLLATE=Swedish_Sweden.1252  LC_CTYPE=Swedish_Sweden.1252
## [3] LC_MONETARY=Swedish_Sweden.1252 LC_NUMERIC=C
## [5] LC_TIME=Swedish_Sweden.1252
##
## attached base packages:
## [1] grid      stats     graphics  grDevices utils     datasets  methods
## [8] base
##
## other attached packages:
##  [1] piecewiseSEM_2.0.2 lme4_1.1-18-1      Matrix_1.2-14
##  [4] multcomp_1.4-8     TH.data_1.0-9      MASS_7.3-50
##  [7] mvtnorm_1.0-8      nlme_3.1-137       contrast_0.21
## [10] rms_5.1-2          SparseM_1.77       Hmisc_4.1-1
## [13] ggplot2_3.1.0.9000 Formula_1.2-3      survival_2.43-1
## [16] lattice_0.20-35    knitr_1.21
##
## loaded via a namespace (and not attached):
##  [1] splines_3.5.0       carData_3.0-1       assertthat_0.2.0
##  [4] highr_0.7           latticeExtra_0.6-28 cellranger_1.1.0
##  [7] pillar_1.3.1        backports_1.1.2     quantreg_5.36
## [10] glue_1.3.0.9000     digest_0.6.18       RColorBrewer_1.1-2
## [13] checkmate_1.8.5     minqa_1.2.4         colorspace_1.4-0
## [16] sandwich_2.5-0      htmltools_0.3.6     pkgconfig_2.0.2
## [19] haven_1.1.2         purrr_0.3.0         scales_1.0.0
## [22] openxlsx_4.1.0      rio_0.5.10          MatrixModels_0.4-1
## [25] htmlTable_1.12      tibble_2.0.1        car_3.0-0
## [28] withr_2.1.2         nnet_7.3-12         lazyeval_0.2.1
## [31] pbkrtest_0.4-7      readxl_1.1.0        magrittr_1.5
## [34] crayon_1.3.4        polspline_1.1.13    evaluate_0.11
## [37] forcats_0.3.0       foreign_0.8-70      tools_3.5.0
## [40] data.table_1.12.0   hms_0.4.2           stringr_1.3.1
## [43] munsell_0.5.0       zip_1.0.0           cluster_2.0.7-1
## [46] bindrcpp_0.2.2      compiler_3.5.0      tinytex_0.6
## [49] rlang_0.3.1         nloptr_1.0.4        rstudioapi_0.7
## [52] htmlwidgets_1.2     base64enc_0.1-3     gtable_0.2.0
## [55] codetools_0.2-15    abind_1.4-5         curl_3.2
## [58] R6_2.2.2            gridExtra_2.3       zoo_1.8-3
## [61] dplyr_0.7.8         bindr_0.1.1         stringi_1.2.4
## [64] parallel_3.5.0      Rcpp_1.0.0          rpart_4.1-13
```

```
## [67] acepack_1.4.1       tidyselect_0.2.5    xfun_0.3
```
